# Delta Opioid Receptors within the Cortico‐Thalamic Circuitry Underlie Hyperactivity Induced by High‐Dose Morphine

**DOI:** 10.1002/advs.202503831

**Published:** 2025-11-30

**Authors:** Chun‐Yue Li, Huiqian Huang, Xiao‐Fan Shen, Ke‐Lei Cao, Di Zheng, Yi Zhu, Shi‐Ze Xie, Xiao‐Dan Yu, Hao Wang, Jia‐Dong Chen, Jie Shi, Yue Li, Min Yan, Xiao‐Ming Li

**Affiliations:** ^1^ Department of Psychiatry of the Second Affiliated Hospital and Liangzhu Laboratory Zhejiang University School of Medicine Hangzhou 310058 China; ^2^ Department of Anesthesiology of Second Affiliated Hospital Zhejiang University School of Medicine Hangzhou 310058 China; ^3^ NHC and CAMS Key Laboratory of Medical Neurobiology Ministry of Education Frontier Science Center for Brain Research and BrainMachine Integration School of Brain Science and Brain Medicine Zhejiang University Hangzhou 310058 China; ^4^ Affiliated Mental Health Center and Hangzhou Seventh People's Hospital Zhejiang University Hangzhou 310013 China; ^5^ State Key Laboratory of Medical Neurobiology and MOE Frontiers Center for Brain Science Institutes of Brain Science Fudan University Shanghai 200032 China; ^6^ Research Units for Emotion and Emotion disorders Chinese Academy of Medical Sciences Center for Brain Science and Brain‐Inspired Intelligence Beijing 100730 China; ^7^ National Institute on Drug Dependence and Beijing Key Laboratory of Drug Dependence Peking University Beijing 100191 China

**Keywords:** cingulate cortex, delta opioid receptors, hyperactivity, morphine, zona incerta

## Abstract

Hyperactivity is a well‐documented neurobehavioral effect of morphine and other opioid drugs, predominantly observed in rodent models, yet the neural circuits and molecular mechanisms underlying this effect remain elusive. In this study, an excitatory projection from the cingulate cortex (Cg) to the intermediate rostrocaudal division of zona incerta (ZIm) is revealed that is activated by morphine in mice. Chemogenetic inhibition of the Cg‐ZIm pathway decreased high‐dose (10–15mg kg^−1^) morphine‐induced hyperlocomotion without affecting its analgesic effects. Activation of this pathway faithfully reproduced the motor effect of morphine. Furthermore, high‐dose morphine‐induced hyperlocomotion is quickly attenuated by microinjecting delta‐opioid receptor (DOR) antagonists into the ZI, which is not observed following the targeted knockout of the DOR in Cg‐projecting ZI neurons, indicating a postsynaptic DOR‐mediated mechanism. In summary, these findings identify the critical role of the DOR within the Cg‐ZIm circuit in the psychomotor properties of morphine. This work sheds light on potential targets within the Cg‐ZIm pathway for mitigating the undesired psychomotor effects of morphine and thereby optimizing its clinical outcomes.

## Graphical abstract

Morphine activates the excitatory cingulate cortex–intermediate rostrocaudal division of zona incerta (Cg‐ZIm) pathway to drive hyperlocomotion in mice. Inhibiting the Cg‐ZIm pathway attenuates both acute and chronic morphine‐induced hyperlocomotion, while its activation mimics morphine's motor effects. Furthermore, delta‐opioid receptors (DOR) within the ZIm are required for this response, revealing a key circuit and receptor mechanism underlying high‐dose morphine‐induced psychomotor activation.

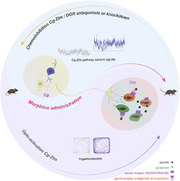

## 1 Introduction

Locomotor activity has been deemed an indicator in predicting the reinforcing properties of a drug.^[^ ^1^, ^2^ ^]^ Nevertheless, although hyperlocomotion is a well‐documented consequence of morphine administration, the neural circuit and molecular mechanisms underlying this effect remain unknown. A recent study has identified the rostral ventral tegmental area (rVTA)‐dorsal raphe nucleus (DRN) pathway as a critical modulator of morphine reward, leaving its analgesic effects and hyperlocomotion unaffected.^[^ ^3^ ^]^ Therefore, understanding how distinct circuits engage in different behavioral adaptations induced by morphine is an important question awaiting future study.

A growing body of evidence indicates that opioids exert potent effects brain‐wide, including the frontal cortex and thalamus.^[^ ^4^, ^5^, ^6^ ^]^ The zona incerta (ZI), a subthalamic region composed of over 85% GABAergic neurons,^[^ ^7^ ^]^ has been implicated as a central hub in regulating pain, defensive behaviors, sensorimotor gating, and motor control.^[^ ^7^, ^8^, ^9^, ^10^, ^11^, ^12^, ^13^, ^14^, ^15^, ^16^, ^17^, ^18^, ^19^, ^20^, ^21^, ^22^, ^23^, ^24^, ^25^, ^26^ ^]^ Although all three major opioid receptors—mu‐, delta‐, and kappa‐opioid receptors (MOR, DOR, KOR)—are expressed in the ZI,^[^ ^27^, ^28^, ^29^, ^30^ ^]^ its contribution to opioid‐induced behavioral responses remains unexplored. The ZI is anatomically and functionally heterogeneous, and its subdivisions (rostral, intermediate rostrocaudal division, caudal; dorsal vs. ventral) differ markedly in molecular identity, input‐output connectivity, and behavioral relevance. The zona incerta, intermediate rostrocaudal division (ZIm) integrates multimodal sensory inputs and exerts GABAergic control over higher‐order thalamic nuclei, positioning it as a key node in the regulation of sensorimotor processing and locomotor behavior.^[^ ^18^, ^19^, ^31^ ^]^ Given its functional connectivity and involvement in motor control,^[^ ^16^, ^23^, ^24^, ^25^ ^]^ ZI has emerged as a promising target for clinical neuromodulation, including deep brain stimulation (DBS) for tremor relief.^[^ ^26^, ^32^, ^33^, ^34^ ^]^

The cingulate cortex (Cg) is anatomically positioned to influence distributed brain networks via its top‐down projections and thus is postulated to broadly regulate behaviors.^[^ ^35^ ^]^ Previous studies of Cg and its associated circuits have unveiled its diverse roles in high‐level cognitive processes and pain‐related affect.^[^ ^36^, ^37^, ^38^ ^]^ McDevitt and colleagues demonstrated that opioid withdrawal altered neuronal properties within the Cg, yet the involvement of Cg in opioid‐induced hyperlocomotion remains unclear.^[^ ^39^ ^]^ Given prior anatomical evidence that the Cg projects directly to the ZI,^[^ ^40^, ^41^, ^42^, ^43^ ^]^ we propose that this pathway may contribute to the top‐down regulation of morphine‐induced hyperlocomotion.

In this study, using c‐Fos brain mapping, fiber photometry, and circuit‐specific optogenetic and chemogenetic approaches, we identify an excitatory projection from the Cg to the ZIm that plays a critical role in high‐dose morphine‐induced psychomotor activation. Additionally, local infusion of the DOR antagonist into the ZIm prevented high‐dose morphine‐induced hyperlocomotion. This effect was blocked by the specific knockout of DOR in Cg‐projecting ZIm neurons. Our findings identify a circuit mechanism that may underlie opioid‐induced psychomotor activation in rodents—a preclinical correlate of motivational and reinforcing properties—and suggest potential targets for mitigating the neural adaptations associated with opioid exposure.

## 2 Results

### 2.1 The Vglut2^+^ Cg Neurons Primarily Innervate ZIm

**Figure 1:**
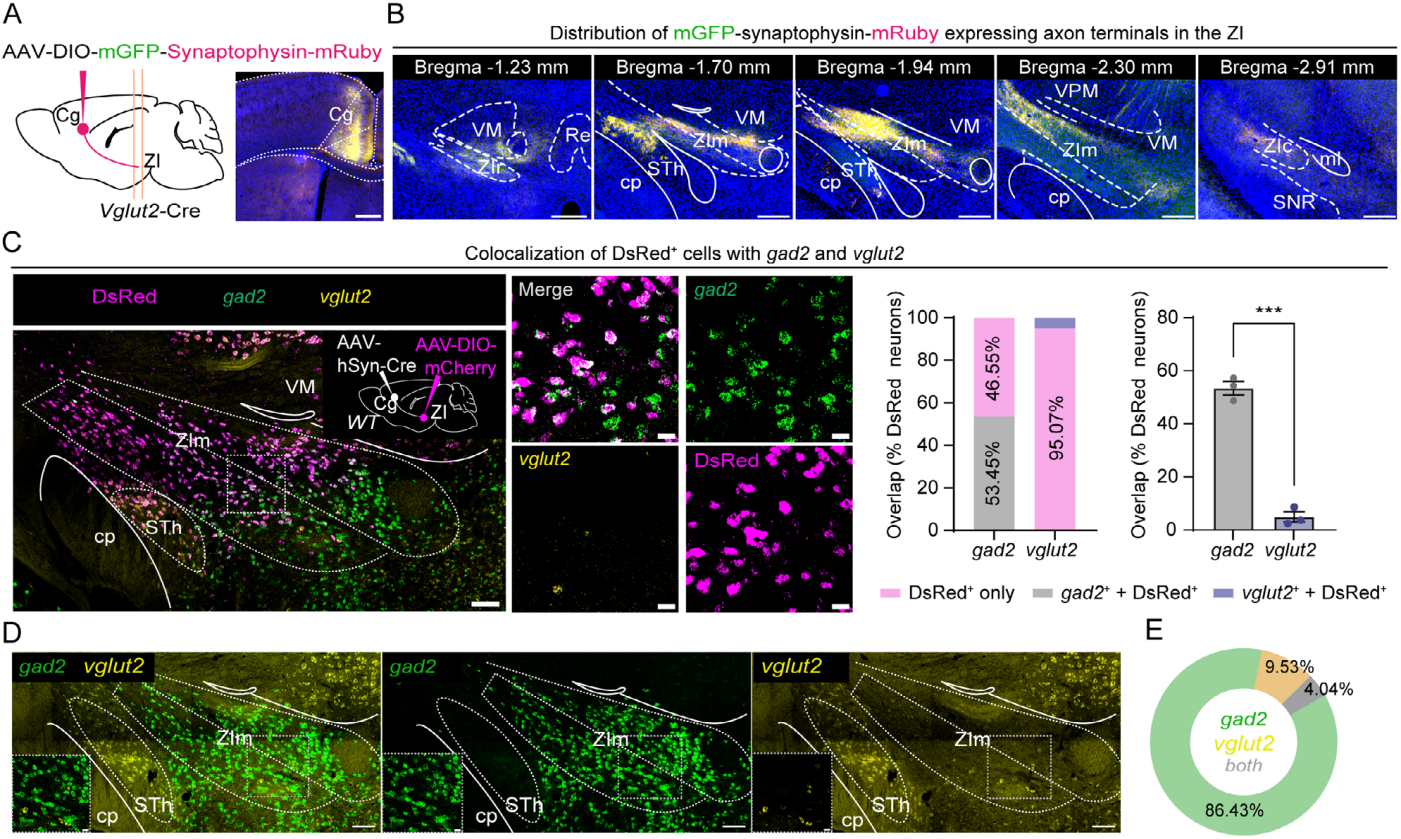
Cg glutamatergic neurons synapse onto ZIm neurons. A) Schematic diagram and example coronal brain sections showing injections of AAV‐DIO‐mGFP‐Synaptophysin‐mRuby in the Cg of *Vglut2*‐Cre mice. Scale bar, 200 µm. B) Representative images show anterograde labeling with mGFP‐Synaptophysin‐mRuby in the ZI, ranging from Bregma −1.23 to −2.91 mm. Scale bars, 500 µm. C) Example micrographs show circuit‐specific anterograde labeling with DsRed^+^ in the ZIm (left, scale bar, 100 µm) and quantitative analyses (right, scale bar, 20 µm) showing DsRed^+^ cells in the ZIm are predominantly positive for gad2 and almost not co‐labeled with vglut2 (*gad2* ^+^ DsRed^+^/DsRed^+^, 53.45% ± 2.53%; *vglut2* ^+^ DsRed^+^/DsRed^+^, 4.93% ± 1.89%; t_(4)_ = 15.37, ^***^ *P* = 0.0001; two‐sided unpaired t‐test; n =3 mice). D) Example confocal images of ZI‐containing coronal brain slices with the RNAScope labeling of the mRNAs for *gad2* (green) and *vglut2* (yellow), and the merged image. Scale bars, 100 and 20 µm. E) Pie chart quantification of the proportion of cells in ZI expressing gad2, vglut2, or both (*n* = 3 mice). ^***^ *P* < 0.001. Error bars represent s.e.m.

Previous studies have characterized the topographic organization of the Cg–ZI pathway.^[^ ^37^, ^40^, ^41^, ^43^, ^44^ ^]^ To further delineate the synaptic connections between the Cg and ZI, we unilaterally injected AAV2/9‐DIO‐mGFP‐Synaptophysin‐mRuby, an anterograde and non‐transsynaptic tracer, into the Cg of *Vglut2*‐Cre mice to visualize axon terminals of Cg glutamatergic neurons (**Figure** **1**A) and found the mGFP and synaptophysin‐mRuby positive terminals mainly located at the ZIm (bregma −1.70 to −2.30 mm) (Figure 1B). We retrogradely labeled Cg neurons projecting to the ZIm by injecting Alexa Fluor 555‐conjugated cholera toxin subunit B (recombinant) (CTB‐555) into the ZIm of wild‐type (WT) mice (Figure S1A,B, Supporting Information), and found retrogradely labeled cells (CTB‐555^+^) in the Cg were distributed from anterior to posterior (Figure S1C, Supporting Information). Additional CTB experiments (Figure S2, Supporting Information) were performed to compare inputs across multiple rostrocaudal tiers of the ZI, including the rostral (ZIr), intermediate rostrocaudal division (ZIm), and caudal (ZIc) subdivisions. Quantitative analysis revealed that the Cg sends only sparse projections to the caudal ZI (ZIc) (Figure S2I–L, Supporting Information). In contrast, the vast majority of retrogradely labeled neurons in the Cg simultaneously projected to both the medial and lateral parts of the ZIm (Figure S2A–D, Supporting Information).

To identify the neuronal subtypes in the ZIm that received Cg innervation, we first employed immunohistochemistry (IHC) to detect the colocalization of mGFP‐Synaptophysin‐mRuby axon terminals with GABA and glutamate within the ZIm. The results revealed that ≈50% of GABAergic neurons in the ZIm colocalized with mGFP‐Synaptophysin‐mRuby axon terminals (Figure S1F,G, Supporting Information), while around 20% of glutamatergic neurons in the ZIm showed colocalization (Figure S1D,E, Supporting Information). To further verify these synaptic connections using an independent approach, we then injected the AAV2/1‐Cre into the Cg and AAV2/9‐DIO‐mCherry into the ipsilateral ZIm of WT mice. AAV2/1‐hSyn‐Cre, a serotype that has been shown to support anterograde, monosynaptic transfer of Cre protein from presynaptic to postsynaptic neurons. The Cre‐transport efficiency has been previously validated in several studies^[^ ^45^, ^46^, ^47^, ^48^ ^]^ and has become a widely accepted approach for tracing anatomical projections and inducing Cre‐dependent expression in downstream targets. Triple‐labeling in situ hybridization of *gad2*, DsRed (dsRed probe was used to detect mCherry mRNA expression),^[^ ^49^, ^50^ ^]^ and *vglut2* mRNA showed that 50% of DsRed‐mRNA‐positive neurons in the ZIm overlapped with *gad2* mRNA, and only 1% of DsRed‐mRNA‐positive neurons overlapped with *vglut2* mRNA (Figure 1C). We quantified the relative proportions of GABAergic and glutamatergic neurons in the ZIm by labeling the canonical markers gad2 and vglut2, respectively. Our analysis revealed that *gad2*‐expressing neurons were markedly more abundant than *vglut2*‐expressing neurons (*gad2*‐only, 86.4% and *vglut2*‐only, 9.5%; Figure 1D,E). These results are consistent with previous characterizations of ZI cell‐type composition.^[^ ^7^, ^51^ ^]^ Importantly, this cellular distribution supports the interpretation that glutamatergic inputs from the Cg predominantly target GABAergic neurons within the ZIm, highlighting a circuit architecture favoring top‐down inhibitory control.

To reinforce the anterograde tracing study and characterize cell‐type‐specific monosynaptic inputs from the Cg to the ZIm, we employed a retrograde viral tracing strategy combined with Cre‐dependent labeling. We injected a mixture of helper viruses (rAAV2/9‐EF1α‐DIO‐N2cG and rAAV2/9‐EF1G‐DIO‐EGFP‐T2A‐TVA, 1:1) into the ZIm of *Gad2*‐Cre and *Vglut2*‐Cre mice because the ZIm consists of a dominant population of GABAergic neurons and a small subset of glutamatergic neurons^[^ ^51^ ^]^ (Figure 1D,E) followed by the envelope protein from avian ASLV type A (EnvA)‐pseudotyped and rabies virus glycoprotein (RVG)‐deleted rabies virusCVS‐EnvA‐AG‐ldTomato at the same coordinates 2 weeks later as reported in previous research.^[^ ^3^, ^52^, ^53^, ^54^ ^]^ Neurons co‐expressing the RVG and TVA (starter cells) permitted monosynaptic retrograde transmission of the rabies virus to presynaptic neurons (input cells) (Figure S1I,L, Supporting Information). In both *Gad2*‐Cre and *Vglut2*‐Cre mice, we observed dsRed‐labeled neurons in the Cg (Figure S1J,K,M,N, Supporting Information), Subsequently, IHC staining with anti‐gaba and anti‐glutamate antibodies was conducted in the Cg. Immunofluorescence staining analysis demonstrated that 58.14% of labeled neurons were glutamate positive and 0.98% were GABA positive in *Gad2*‐Cre mice, while 51.31% of labeled neurons were glutamate positive and 2.59% were GABA positive in *Vglut2*‐Cre mice (Figure S1J,K,M,N, Supporting Information).

Our data demonstrate that glutamatergic neurons from the Cg form dense projections to the ZIm, preferentially targeting gad2^+^ neurons over glutamatergic ones.

### 2.2 Morphine Increases Neuronal Activity in the Cg‐ZIm Pathway

**Figure 2:**
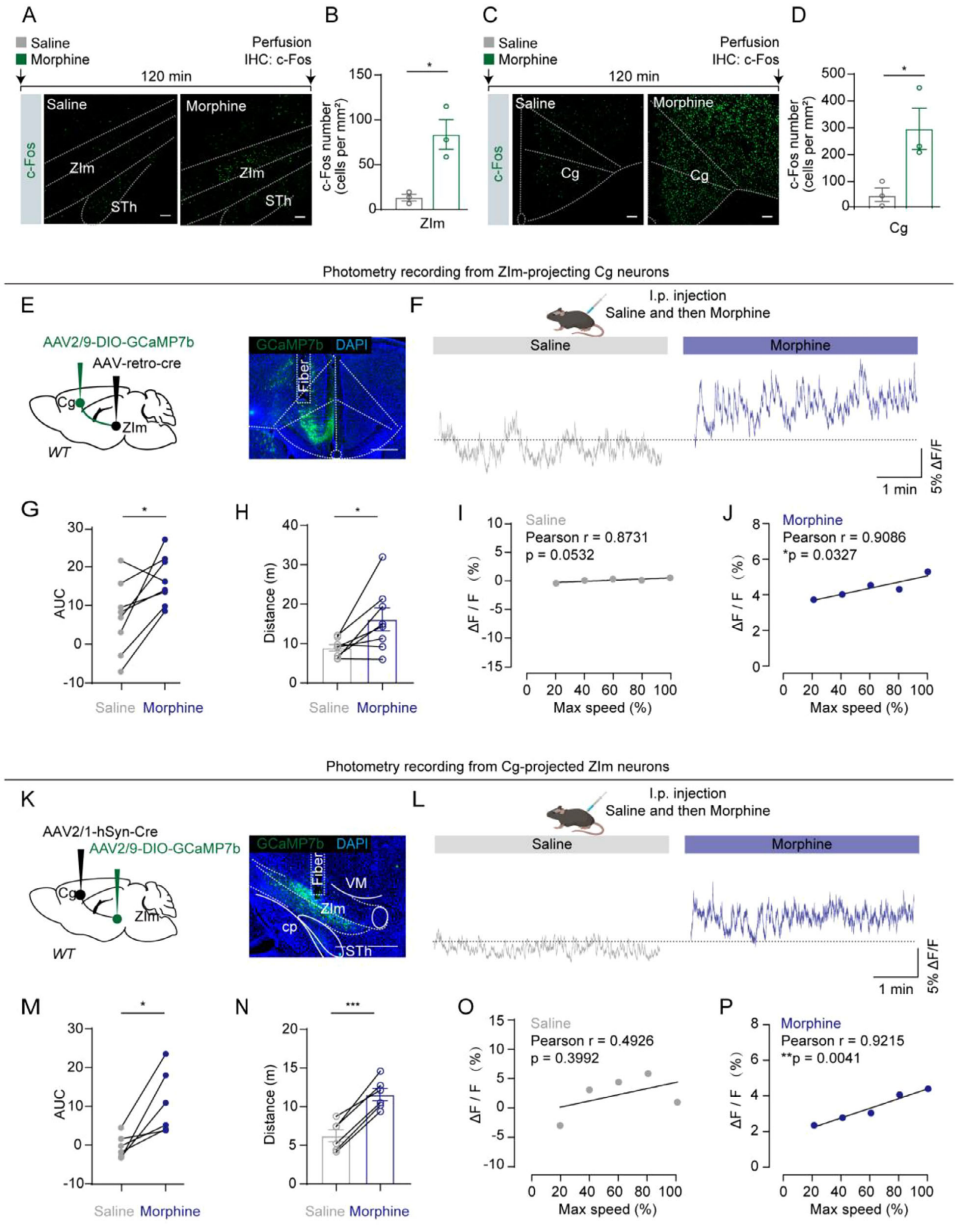
The Cg‐ZIm circuit is involved in morphine‐induced hyperlocomotion. A, C) Experimental paradigm and representative images of c‐fos staining. Scale bars, 100 µm. B, D) The density of c‐Fos expressing neurons in the ZIm (B, t_(4)_ = 4.166, P = 0.0141) and Cg (D, t_(4)_ = 3.033, P = 0.0387). *n* = 3 for each group. E, K) Schematic diagram and an example coronal section (E, showing the optical fiber track above the GCaMP7b^+^ Cg^ZIm^ neurons; K, showing the optical fiber track above the GCaMP7b^+^ Cg^ZIm^ neurons). Scale bars, 500 µm. F, L) Time courses of normalized average GCaMP fluorescence of Cg^ZIm^ neurons in F or ZIm^Cg^ neurons in L from mice receiving saline or morphine injection. G, M) AUC of saline and morphine‐induced signal (G, t_(8)_ = 3.353, P = 0.01; M, t_(7)_ = 4.534, P = 0.0027). H, N) Quantitative analyses of the total distance (H, *left*, t_(14)_ = 2.386, P = 0.0317; N, *left*, t_(14)_ = 4.247, P = 0.0008) during i.p. injections of saline and morphine. I, J) Correlation between normalized average GCaMP fluorescence of Cg^ZIm^ neurons and locomotor speed from mice receiving saline (I) or morphine (J) injection. n (mice) = 8. O, P) Correlation between normalized average GCaMP fluorescence of ZIm^Cg^ neurons and locomotor speed from mice receiving saline (O) or morphine (P) injection. (velocity is binned at intervals of 20% of the maximum speed). n (mice) = 6. ^*^ *P* < 0.05, ^***^ *P* < 0.001, n.s. = no significant difference. Error bars represent s.e.m. B, D, two‐sided unpaired t‐test; G, H, M, N, two‐sided paired t‐test.

Next, we injected mice with morphine (15 mg kg^−1^) or saline intraperitoneally (i.p.) to investigate the effects of morphine on the Cg‐ZIm circuits. We first used c‐Fos expression to examine whether the Cg and ZIm are activated by morphine. Compared to saline controls, morphine markedly increased c‐Fos expression in both regions (**Figure** **2**A–D). In addition, we observed elevated c‐Fos levels in several other brain areas previously reported to be involved in morphine‐related responses (Figure S3, Supporting Information).^[^ ^27^, ^55^, ^56^, ^57^ ^]^ To investigate whether the increased c‐Fos expression indicated increased neuronal activity, we recorded the population activity of the Cg‐ZIm pathway in WT mice after systematic administration of morphine. To achieve this, we expressed GCaMP7b in either Cg neurons projecting to the ZIm (Cg^ZIm^) or ZIm neurons downstream of the Cg (ZIm^Cg^) by either delivering the rAAV2/2‐Retro‐Cre into the ZIm and AAV2/9‐DIO‐GCaMP7b into the ipsilateral Cg (Figure 2E) or injecting AAV2/1‐Cre into the Cg and AAV2/9‐DIO‐GCaMP7b into the ipsilateral ZIm (Figure 2K). After recovery from the surgeries, animals were placed in an experimental cage and GCaMP7b fluorescence indicating neuronal activity was recorded for 5 min, starting 5 min after i.p. injections of first saline and then morphine. GCaMP7b fluorescence recordings showed that morphine increased neural activities of the Cg‐ZIm pathway (Figure 2F,G,L,M) together with increasing the locomotor activity of mice (Figure 2H,N) compared to saline. Furthermore, the elevation of calcium signals was significantly correlated with the velocity of mice upon morphine administration of morphine (Figure 2I,J,O,P). These data indicate that Cg‐ZIm pathways are recruited in morphine‐induced hyperactivity.

### 2.3 The Cg‐ZIm Pathway is Required for Morphine‐Induced Hyperlocomotion Under High Doses

**Figure 3:**
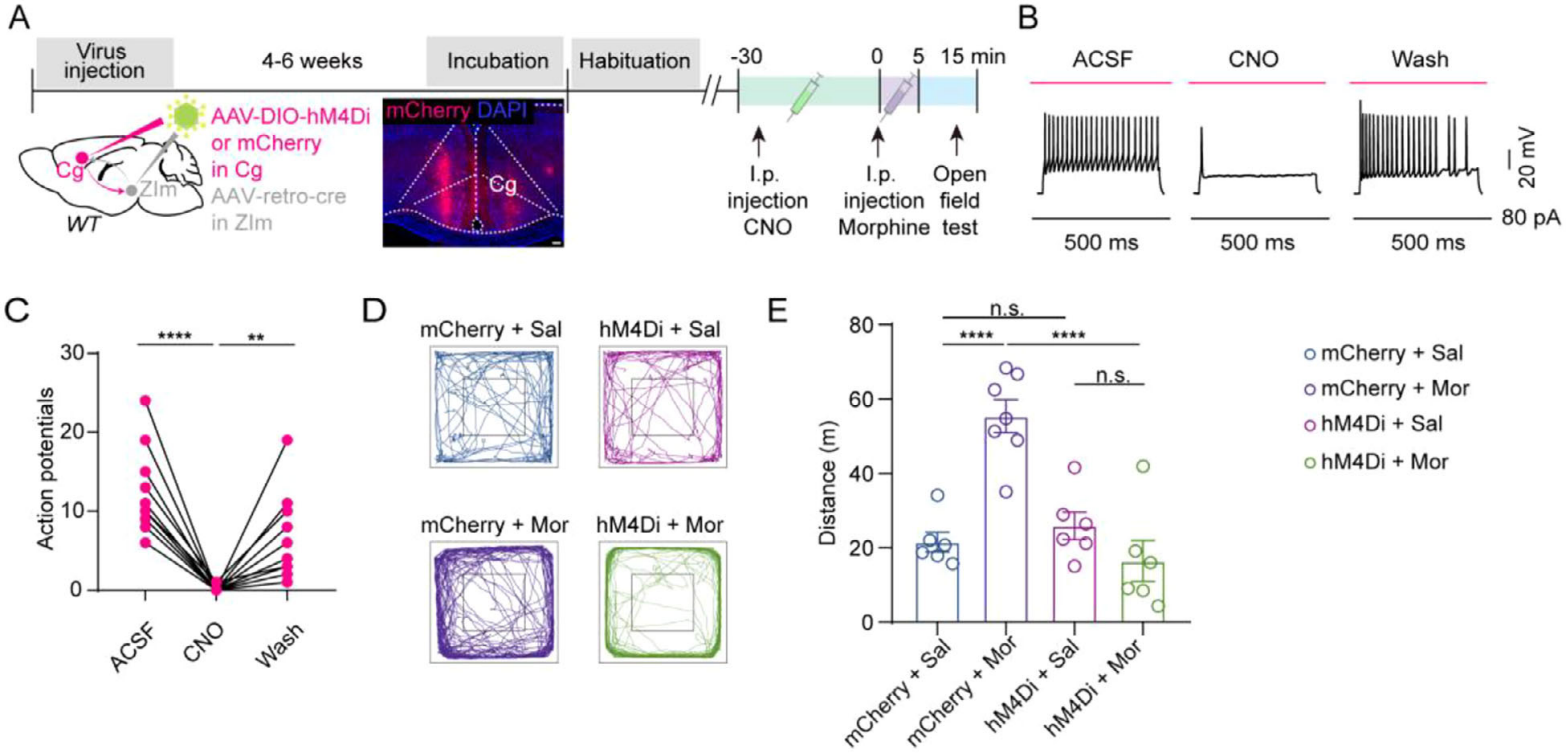
Chemogenetic inhibition Cg‐ZIm pathway diminishes acute morphine hyperlocomotion. A) Experimental and schematic diagram showing injection of AAV‐DIO‐hM4Di‐mCherry and AAV‐Retro‐Cre into the Cg and ZIm of WT mice. Scale bars, 200 µm. B, C) Current‐evoked action potentials in a representative hM4Di‐infected Cg presynaptic neuron recorded before, during, and after CNO (10 mM) perfusion (F_(1.130, 11.30)_ = 41.70, ^****^ *P* < 0.0001; ^****^ *P*_ACSF vs. CNO_ < 0.0001; ^**^ *P*_CNO vs. Wash_ = 0.0066; two‐sided one‐way ANOVA with Sidak test). D) Representative track plots after i.p. injections. E) Distance traveled (F_(3, 21)_ = 18.11, ^****^ *P* < 0.0001; ^****^ *P*_mCherry+Sal vs. mCherry+Mor_ < 0.0001; **** P _mCherry+Mor vs. hM4Di+Mor_ < 0.0001; P _hM4Di+Sal vs. hM4Di+Mor_ = 0.3598; two‐sided one‐way ANOVA with Holm‐Sidak test) after i.p. injections. n (mice) = 6 (mCherry + Sal), 7 (mCherry + Mor), 6 (hM4Di + Sal), and 6 (hM4Di + Mor). ^**^ *P* < 0.01, ^****^ *P* < 0.0001, n.s. = no significant difference. Error bars represent s.e.m.

To evaluate the contribution of the Cg‐ZIm pathway to the hyperlocomotion behavior induced by acute morphine administration, we specifically inhibited Cg^ZIm^ in the open field test (OFT) by expressing hM4Di in Cg^ZIm^ via bilateral ZIm injection of rAAV2/2‐Retro‐Cre and bilateral Cg injection of AAV2/9‐DIO‐hM4Di‐mCherry. Thirty minutes after clozapine‐N‐oxide (CNO) i.p. injection (4 mg kg^−1^),^[^ ^58^, ^59^ ^]^ mice were systematically administered morphine and left in the arena for another 10 min (**Figure** **3**A). The hM4Di‐mCherry^+^ neurons in the Cg were inhibited by i.p. injection of CNO (Figure 3B,C). Chemogenetic inhibition of Cg^ZIm^ neurons decreased distance in the OFT boosted by morphine (Figure 3D,E). Notably, inhibition of the circuit alone did not affect the movement of mice in the open field (Figure 3E). In addition, we examined the role of the Cg‐ZIm pathway in morphine‐induced locomotion at different doses. Chemogenetic inhibition of the Cg‐ZIm circuit had no significant effect at a low dose (5 mg kg^−1^) but markedly suppressed hyperlocomotion at higher doses (10–15 mg kg^−1^), suggesting a dose‐dependent involvement of the Cg‐ZIm pathway (Figure S4A,B, Supporting Information). Besides, this manipulation did not affect the anxiety‐like behavior in the elevated plus maze test (EPM), morphine‐induced conditioned place preference (CPP), and analgesia in the hot plate test (HPT) (Figure S4C–K, Supporting Information).

To ascertain the role of ZIm^Cg^ in morphine‐induced hyperlocomotion, we selectively inhibited this pathway by delivering the monosynaptic anterograde transport virus AAV2/1‐Cre into the bilateral Cg and AAV2/9‐DIO‐hM4Di‐mCherry into the bilateral ZIm (Figure S5A, Supporting Information). Chemogenetic suppression of ZIm^Cg^ neurons significantly decreased the distance boosted by morphine (Figure S5B, Supporting Information).

Given that ≈50% of ZIm neurons receiving Cg input express gad2, we further investigated the function of this GABAergic population using both optogenetic activation and chemogenetic inhibition. Optogenetic activation of ZIm^gad2^ neurons had no effect on baseline locomotor activity (Figure S6A,B, Supporting Information), indicating that these cells may not regulate spontaneous movement under physiological conditions. To directly assess the role of ZIm^gad2^ neurons under morphine challenge, we performed chemogenetic inhibition in *Gad2*‐Cre mice by bilaterally injecting AAV2/9‐hSyn‐DIO‐hM4Di‐mCherry into the ZIm (Figure S6C, Supporting Information). Four weeks later, mice received systemic injections of morphine at 15 mg kg^−1^ (i.p.), followed 30 min later by administration of the hM4Di agonist CNO (4 mg kg^−1^, i.p.). Locomotor activity recorded over the subsequent 10 min showed that chemogenetic silencing of ZI^gad2^ neurons significantly attenuated morphine‐induced hyperlocomotion (Figure S6D, Supporting Information). To explore the contribution of non‐GABAergic neurons, we next injected a Cre‐off‐4Di virus into the ZIm of *Gad*‐Cre mice (Figure S6E, Supporting Information) and found that inhibition of the non‐Gad2 neurons, which included those gad2‐negative Cg‐projecting ZI neurons, led to a decrease in morphine‐induced hyperlocomotion (Figure S6F, Supporting Information), supporting the idea that both Gad2⁺ and non‐Gad2 ZIm neurons contribute to the psychomotor effects of morphine. Together, these results provide further evidence that ZIm neurons are important for morphine‐induced hyperlocomotion.

**Figure 4:**
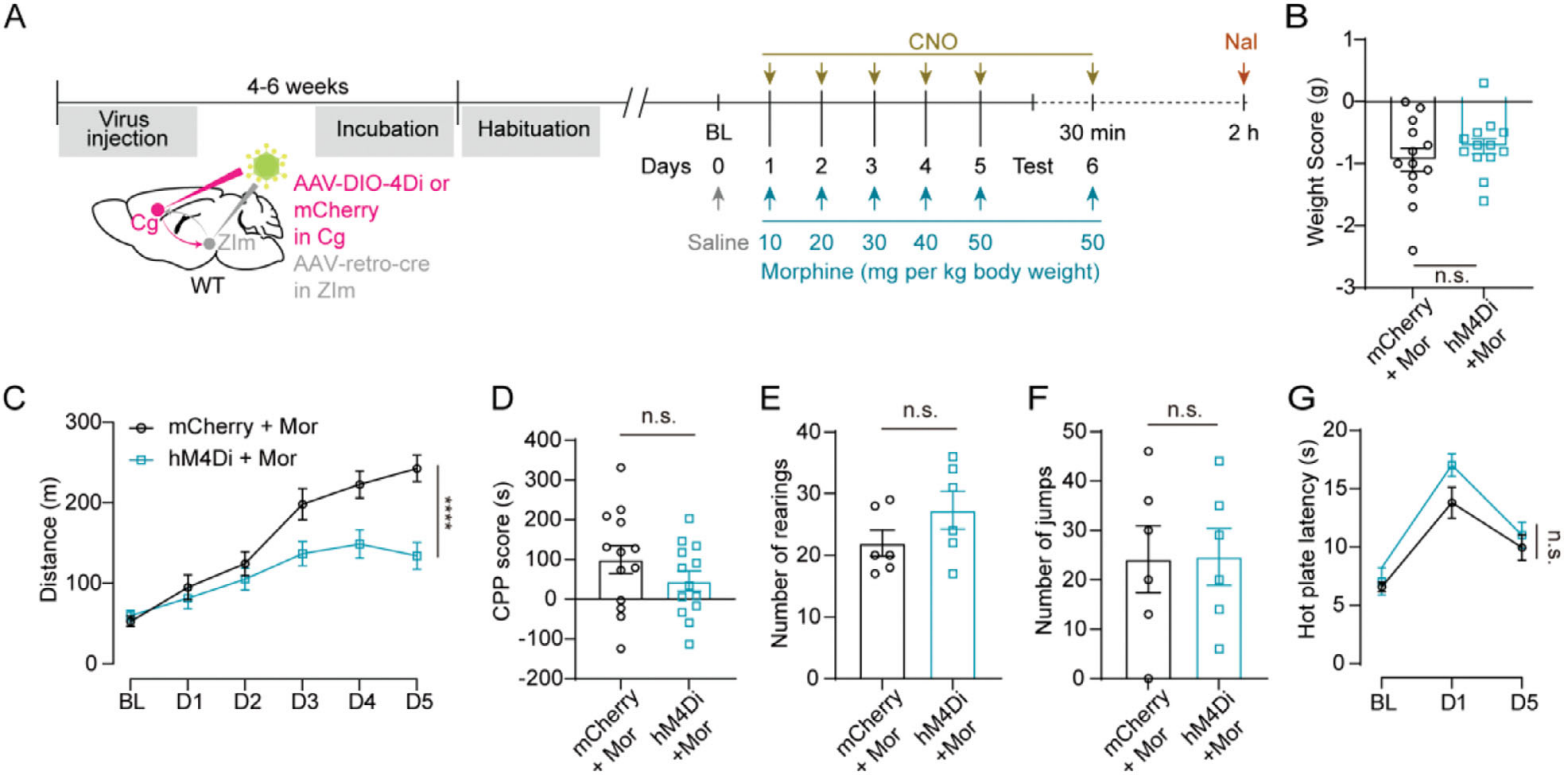
Chemogenetic inhibition Cg‐ZIm pathway selectively diminishes chronic morphine hyperlocomotion. A) Drug administration and behavioral test procedures. B) Body weight score for mice following chronic inhibition of the Cg‐ZIm circuit during morphine treatment (t_(24)_ = 0.9673, P = 0.343, two‐sided unpaired t‐test). n (mice) = 13 (mCherry) and 13 (hM4Di). C) Total distance traveled in response to morphine administration while chronically reducing Cg‐ZIm circuit activity within 1 h (F_(5, 120)_ = 6.365, **** P < 0.0001; two‐sided two‐way ANOVA with Sidak test). n (mice) = 13 (mCherry) and 13 (hM4Di). D) CPP scores for mice following chronic inhibition of the Cg‐ZIm circuit (t_(24)_ = 1.246, P = 0.2248; two‐sided unpaired t‐test). n (mice) = 13 (mCherry) and 13 (hM4Di). (E, F) Rearing (E, t_(10)_ = 1.437, P = 0.1813; two‐sided unpaired t‐test) and jump number (F, t_(10)_ = 0.05637, P = 0.9562; two‐sided unpaired t‐test) after naloxone administration in mice following chronic inhibition of the Cg‐ZIm circuit during morphine treatment. n (mice) = 6 (mCherry) and 6 (hM4Di). G) Hot plate latency on different days for mice following chronic inhibition of the Cg‐ZIm circuit during morphine treatment (F_(2, 20)_ = 0.5248, P = 0.5996; two‐sided two‐way ANOVA with Sidak test). n (mice) = 7 (mCherry) and 5 (hM4Di). ^****^ *P* < 0.0001, n.s. = no significant difference. Error bars represent s.e.m.

Chronic morphine administration induces several other adaptive behaviors, such as behavioral sensitization, addiction, and dependence.^[^ ^60^ ^]^ To further confirm the contribution of the Cg‐ZIm pathway in chronic morphine administration, we chemogenetic inhibited the Cg^ZIm^ in WT animals 30 min before daily injection of morphine with doses escalating from 10 to 50 mg per kg body weight (**Figure** **4**A).^[^ ^3^ ^]^ Chemogenetic inhibition of the Cg^ZIm^ neurons attenuated hyperlocomotion induced by morphine (Figure 4C; Figure S8A, Supporting Information), without affecting mice's body weight (Figure 4B), rewarding effects (Figure 4D), withdrawal symptoms (Figure 4E,F), and analgesic effects of morphine (Figure 4G). These data suggested that the Cg‐ZIm pathway specifically contributed to morphine‐induced hyperlocomotion.

Taken together, these results demonstrate that endogenous activity of the Cg‐ZIm pathway specifically contributes to hyperlocomotion induced by both acute and chronic morphine.

Although GABAergic projections from the Cg to the ZIm represent only a small proportion of the total Cg‐ZIm projection (Figure S1I–N, Supporting Information), they nonetheless warrant further functional investigation. To characterize this pathway, we injected AAV‐DIO‐mGFP into the Cg of *Gad2*‐Cre mice and observed that Cg‐GABAergic neurons sparsely project to both GABAergic and glutamatergic neurons in the ZIm, as confirmed by immunohistochemistry (Figure S7A,B, Supporting Information). To functionally assess the role of this projection, we employed a dual‐recombinase strategy: Retro‐DIO‐Flp was injected into the ZIm and fDIO‐hM4Di into the Cg of *Gad2*‐Cre mice (Figure S7C, Supporting Information), allowing specific labeling and chemogenetic inhibition of Cg‐gad2 neurons projecting to the ZIm. Histological analysis revealed a very sparse population of labeled neurons in the Cg (Figure S7C, Supporting Information), consistent with the limited GABAergic input identified in our anatomical tracing (Figure S1I–N, Supporting Information). Functionally, chemogenetic inhibition of the Cg‐gad2 neurons projecting to the ZIm did not alter morphine‐induced hyperlocomotion (Figure S7D, Supporting Information), suggesting that this projection is not required for the behavioral phenotype under our experimental conditions.

Together, these findings support the conclusion that the Cg^Glu^‐ZIm^GABA^ pathway is the primary functional circuit mediating morphine‐induced hyperlocomotion.

### 2.4 Activation of the Cg‐ZIm Pathway Promotes Locomotion

**Figure 5:**
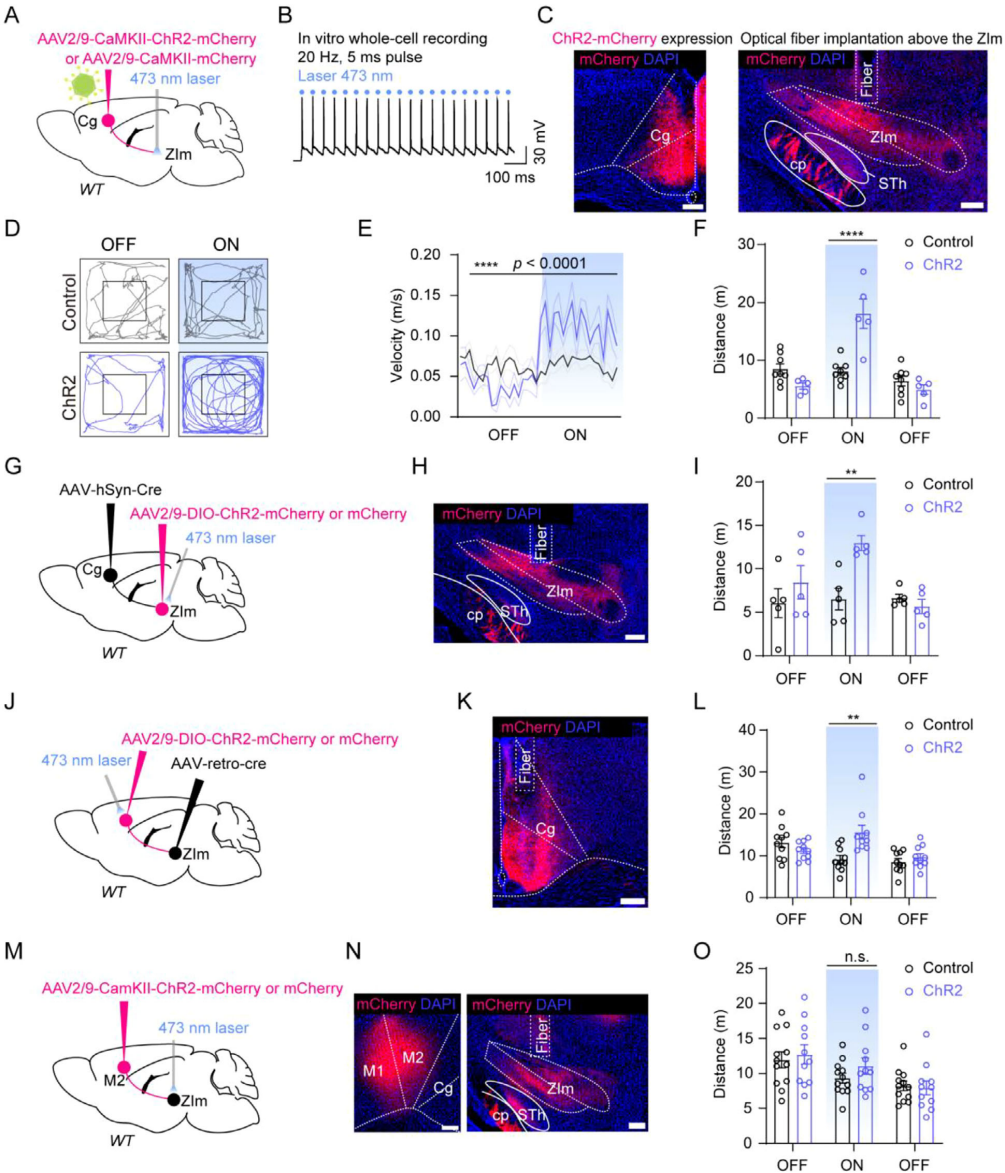
Activation of the Cg–ZIm pathway mimics the effect of morphine injection. A) Schematic of virus injection and optical fiber implantation. B) Electrophysiological verification of ChR2 in ChR2‐mCherry^+^ Cg neuron. C) ChR2 expression (left) and an optical fiber track (right). D–F) Representative tracks D) and statistical analysis (E, F) of optogenetic activation of Cg‐ZIm pathway in the open field test. (E, velocity, F_(35, 385)_ = 4.834, *P* < 0.0001; F, distance, F_(2, 22)_ = 26.96, *P* < 0.0001; *P* < 0.0001 for laser ON stage comparison). n (mice) = 8 (Con) and 5 (ChR2). G, J, M) Experimental details (G, Activation of ZIm^Cg^ neurons; J, Activation of Cg^ZIm^ neurons; M, Activation of M2‐ZIm pathway). H, K, N) ChR2 expression and optical fiber track. I) Quantitative analyses of distance (F_(2, 16)_ = 5.596, P = 0.0144; P = 0.0043 for laser ON stage comparison). n (mice) = 5 (Con) and 5 (ChR2). L) Quantitative analyses of distance (F_(2, 24)_ = 3.725, P = 0.0390; P = 0.0039 for laser ON stage comparison). n (mice) = 6 (Con) and 8 (ChR2). O) Quantitative analyses of distance (F_(2, 20)_ = 1.454, P = 0.2573; P = 0.9467 for laser ON stage comparison). n (mice) = 6 (Con) and 6 (ChR2). ^**^ *P* < 0.01, ^****^ *P* < 0.0001, n.s. = no significant difference. Error bars represent s.e.m. Scale bars, 100 µm. For all figures: two‐sided two‐way ANOVA with Sidak test.

To further explore the role of the Cg‐ZIm pathway in locomotion, we activated this pathway by bilaterally expressing ChR2‐mCherry into the Cg of WT mice and illuminating blue light above the ChR2‐mCherry^+^ axon terminals in the ZIm (**Figure** **5**A,C). In acute brain slices with the Cg, light pulses (20 Hz, 5 ms, 6–8 mW) reliably evoked action potentials from ChR2‐mCherry^+^ Cg neurons (Figure 5B). In the open field tests, photo‐activation of the Cg‐ZIm pathway (5, 10, or 20 Hz, 5 ms, 5 s, 3–5, 6–8 or 8–10 mW) significantly increased the velocity, and total distance of mice (Figure 5E,F; Figure S9A–F, Supporting Information). Moreover, opto‐activation of the Cg‐ZIm pathway in freely moving mice did not affect the time spent in the light‐stimulated chamber in a real‐time place preference (RTPP) paradigm (Figure S10A,B, Supporting Information) or the light‐conditioned chamber in a conditioned place preference (CPP) paradigm (Figure S10C,D, Supporting Information), suggesting that the activation of the Cg‐ZIm pathway was not rewarding. In addition, the opto‐activation of the Cg‐ZIm pathway did not affect anxiety‐like behavior (Figure S10E–H, Supporting Information).

To exclude the possibility that the enhancement of locomotion was due to increased repetitive behavior or an elevation in motor ability,^[^ ^3^ ^]^ we activated the Cg‐ZIm pathway in the marble‐burying test and the rotarod test. The number of buried marbles in the ChR2 group mice was similar to that in the control group (Figure S10I,J, Supporting Information). In the rotarod test, activation of this pathway did not improve motor ability (Figure S10K–M, Supporting Information), suggesting that the hyperlocomotion caused by activation of Cg‐ZIm was not due to enhancement in motor ability.

We further explored whether the activation of ZIm^Cg^ neurons or Cg^ZIm^ can increase locomotion. We specifically expressed ChR2 either in the postsynaptic ZIm neurons by delivering the AAV2/1‐Cre bilaterally into the Cg and AAV2/9‐DIO‐ChR2‐mCherry bilaterally into the ZIm (Figure 5G,H) or in the Cg^ZIm^ by delivering the rAAV2/2‐Retro‐Cre bilaterally into the ZIm and AAV2/9‐DIO‐ChR2‐mCherry bilaterally into the Cg (Figure 5J,K). Activation of either pre‐ or post‐synaptic neurons of the Cg‐ZIm pathway increased locomotion in OFT (Figure 5I,L). As a control, photo‐activation of the projection from the primary motor cortex (M1/M2) to the ZIm showed no significant effect on locomotion behavior (Figure 5M–O). These data indicated that activation of the Cg‐ZIm pathway could trigger hyperlocomotion in WT mice.

### 2.5 Inhibition of Postsynaptic DOR Prevents High‐Dose Morphine‐Induced Hyperlocomotion

**Figure 6:**
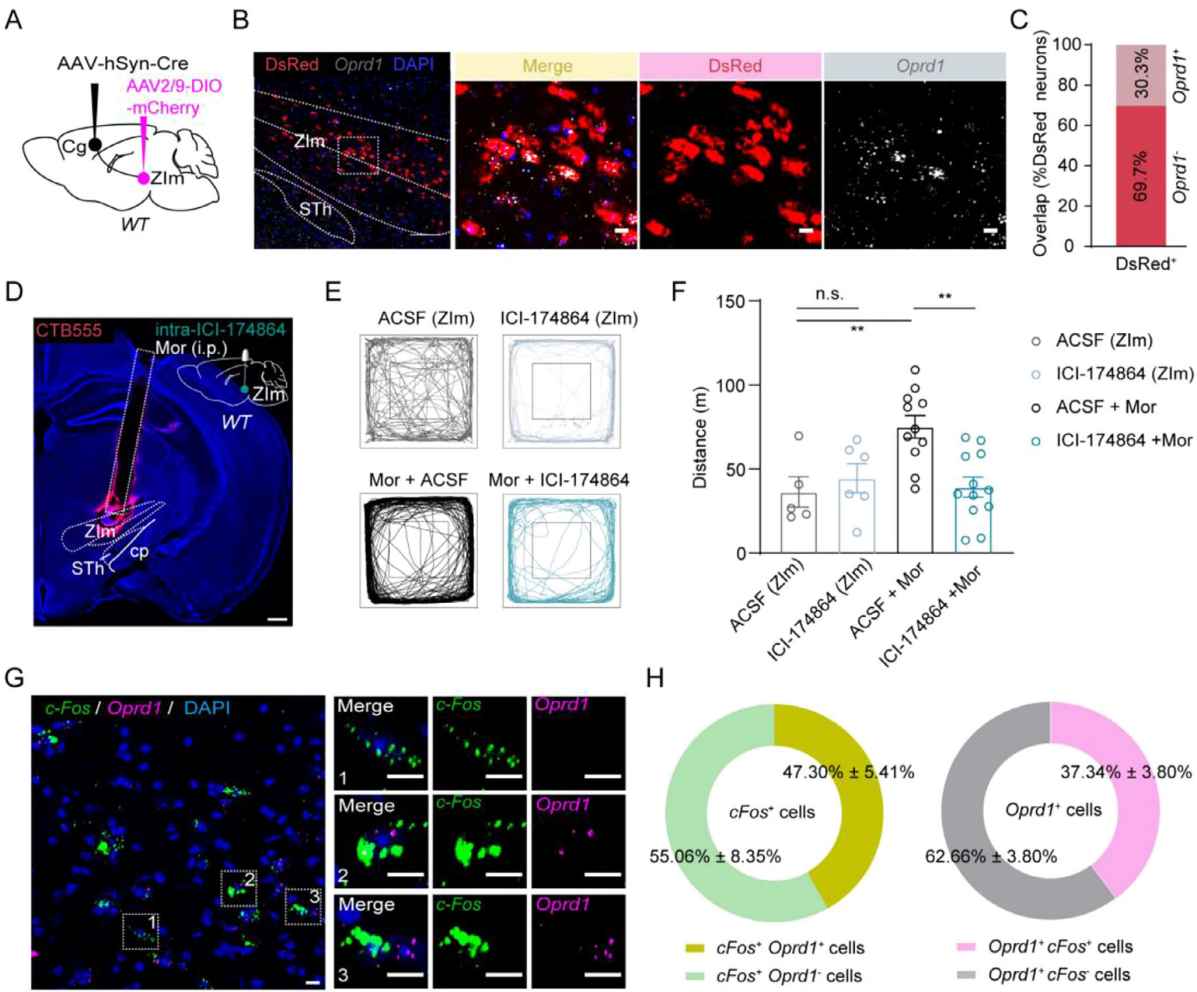
DOR antagonists prevent high‐dose morphine‐induced hyperlocomotion. A) Schematic of virus injection and optical fiber implantation. B) *Left*: a representative image of ZIm showing DsRed‐labeled ZIm that were immunopositive for *Oprd1*. Scale bars, 100 µm. *Right*: magnified image shows the boxed area; scale bar, 10 µm. C) Quantification of co‐localization between DsRed‐ and *Oprd1*‐positive neurons in ZIm (*n* = 3 mice). (DsRed ^+^ *Oprd1* ^+^/DsRed ^+^, 35.88% ± 3.19%; DsRed^+^ *Oprd1* ^−^/DsRed ^+^, 82.24% ± 5.76%). D) Schematic paradigm of drug administration and representative images of CTB555 infection and cannula implantation. Scale bars, 500 µm. E) Representative tracks for saline or morphine without ICI‐174864 and with ICI‐174864 (3 µm). F) Statistics of locomotor distance (F_(3, 30)_ = 7.016, ^**^ *P*=0.0010; P _ACSF vs. ICI174864_ = 0.9889; ^**^ *P*_ACSF+Mor vs. ICI174864+Mor_ = 0.0019; ^**^ *P*_ACSF vs. ACSF+Mor_ = 0.0098; two‐sided one‐way ANOVA with Sidak test). n (mice) = 5 (ACSF‐ZIm), 6 (ICI‐174864‐ZIm), 11 (ACSF‐ZIm + Mor) and 12 (ICI‐174864‐ZIm + Mor). G) Representative images for morphine‐induced c‐Fos expression co‐staining with opioid receptors DOR in ZIm. Insets, high‐magnification micrographs showing c‐Fos‐expression only (1), co‐expression (2), and *Oprd1*‐expression only (3) neurons. Scale bars, 10 µm. H) Quantitative analyses for the percentage of *Oprd1* or *c‐Fos*‐expressing neurons in ZIm. Results are from n = 3 mice. (*left*, *c‐Fos* ^+^ *Oprd1* ^+^/*c‐Fos* ^+^, 47.30% ± 5.41%; *c‐Fos* ^+^ *Oprd1* ^−^/*c‐Fos* ^+^, 55.06% ± 8.35%; *right*, *Oprd1* ^+^ *c‐Fos* ^+^/*Oprd1* ^+^, 37.34% ± 3.80%; *Oprd1* ^+^ *c‐Fos* ^−^/*Oprd1* ^+^, 62.66% ± 3.80%). ^**^ *P* < 0.01, n.s. = no significant difference. Error bars represent s.e.m.

Morphine has the highest affinity to MOR,^[^ ^61^ ^]^ while research suggests DOR may be involved in the psychomotor properties of morphine.^[^ ^62^ ^]^ Given that the studies indicate the presence of three opioid receptors within the ZI,^[^ ^27^, ^28^, ^29^, ^30^ ^]^ to explore the function of these receptors, we first verified their expression in the ZIm.We injected the AAV2/1‐Cre into the Cg and AAV2/9‐DIO‐mCherry into the ipsilateral ZIm (**Figure** **6**A; Figure S11, Supporting Information). According to the data obtained from in situ hybridization and immunofluorescence staining, we observed that around 30.3% of the cells expressing DsRed were immunopositive for *Oprd1*, which encodes the delta‐opioid receptor (DOR) (Figure 6B,C). Similarly, ≈54.1% or 21.3% of the cells expressing DsRed were immunopositive for *oprm1* (encodes the mu‐opioid receptor, MOR) and *oprk1* (encodes the kappa‐opioid receptor, KOR) (Figure S11B–E, Supporting Information).

According to our previous observation that local administration of morphine into the ZIm significantly increased locomotion in mice (Figure S13D, Supporting Information), we next investigated whether this psychomotor effect of morphine was mediated by opioid receptors within the ZIm. To address this, we examined the effects of different doses of morphine and their modulation by pharmacological manipulation. At a low dose (5 mg kg^−1^), we found that the MOR antagonist (β‐FNA, 3 µm) significantly attenuated morphine‐induced hyperlocomotion (Figure S11F,G, Supporting Information), suggesting that MOR is the predominant mediator of morphine‐induced hyperlocomotion at lower concentrations. In line with this, chemogenetic inhibition of the Cg‐ZIm pathway did not significantly reduce hyperlocomotion at low doses (Figure S4B, Supporting Information), indicating that this top‐down cortical‐thalamic circuit is not critically engaged under low‐dose conditions. In contrast, at a higher morphine dose (15 mg kg^−1^), we observed a robust behavioral effect of both DOR (ICI174864, 3 µm) antagonism and chemogenetic inhibition of the Cg‐ZIm circuit. Specifically, the involvement of the Cg‐ZIm projection at high doses (Figure S4B, Supporting Information), and its sensitivity to DOR blockade (Figure 6D–F; Figure S11F,G, Supporting Information), suggests that DORs expressed within this pathway—possibly at the level of ZIm neurons—contribute significantly to the exaggerated psychomotor response. Furthermore, we have observed that ≈47.9% of the *c‐Fos* ^+^ neurons in ZIm, which were activated by morphine, also express *Oprd1*, and ≈36.6% of the *Oprd1* ^+^ neurons within ZIm were also activated by morphine (Figure 6G,H). We also quantified the overlap between *c‐Fos* ^+^ neurons and other opioid receptors, including *Oprm1 (c‐Fos* ^+^ *Oprm1* ^+^/ *c‐Fos* ^+^, 31.4%; *Oprm1* ^+^ *c‐Fos* ^+^/ *Oprm1* ^+^, 18.63%) and *Oprk1 (c‐Fos* ^+^ *Oprk1* ^+^/ *c‐Fos* ^+^, 35%; *Oprk1* ^+^ *c‐Fos* ^+^/ *Oprk1* ^+^, 13.25%) within the ZIm (Figure S11H–K, Supporting Information). This discovery further solidifies the pivotal role of DOR within ZIm in mediating the psychomotor properties of morphine.

To determine whether opioid receptors are expressed in gad2⁺ or gad2^−^ neurons within the ZIm, we performed double‐label RNAscope for gad2 and *Oprd1*, *Oprm1*, or *Oprk1* mRNAs. Quantification across three animals revealed that 53.4 ± 2.5% of *gad2*⁺ neurons co‐expressed *Oprd1*, 25.5 ± 2.8% co‐expressed *Oprm1*, and 26.2 ± 1.8% co‐expressed *Oprk1*. Conversely, 84.63% ± 1.57% of *Oprd1*⁺ neurons, 52.46% ± 3.27% of *Oprm1*⁺ neurons, and 59.28% ± 2.60% of *Oprk1*⁺ neurons co‐expressed *gad2* (Figure S12, Supporting Information). These results suggest that DOR are more enriched in GABAergic (gad2⁺) neurons, while MOR and KOR exhibit less co‐expression with GABAergic (gad2⁺) populations within the ZIm.

**Figure 7:**
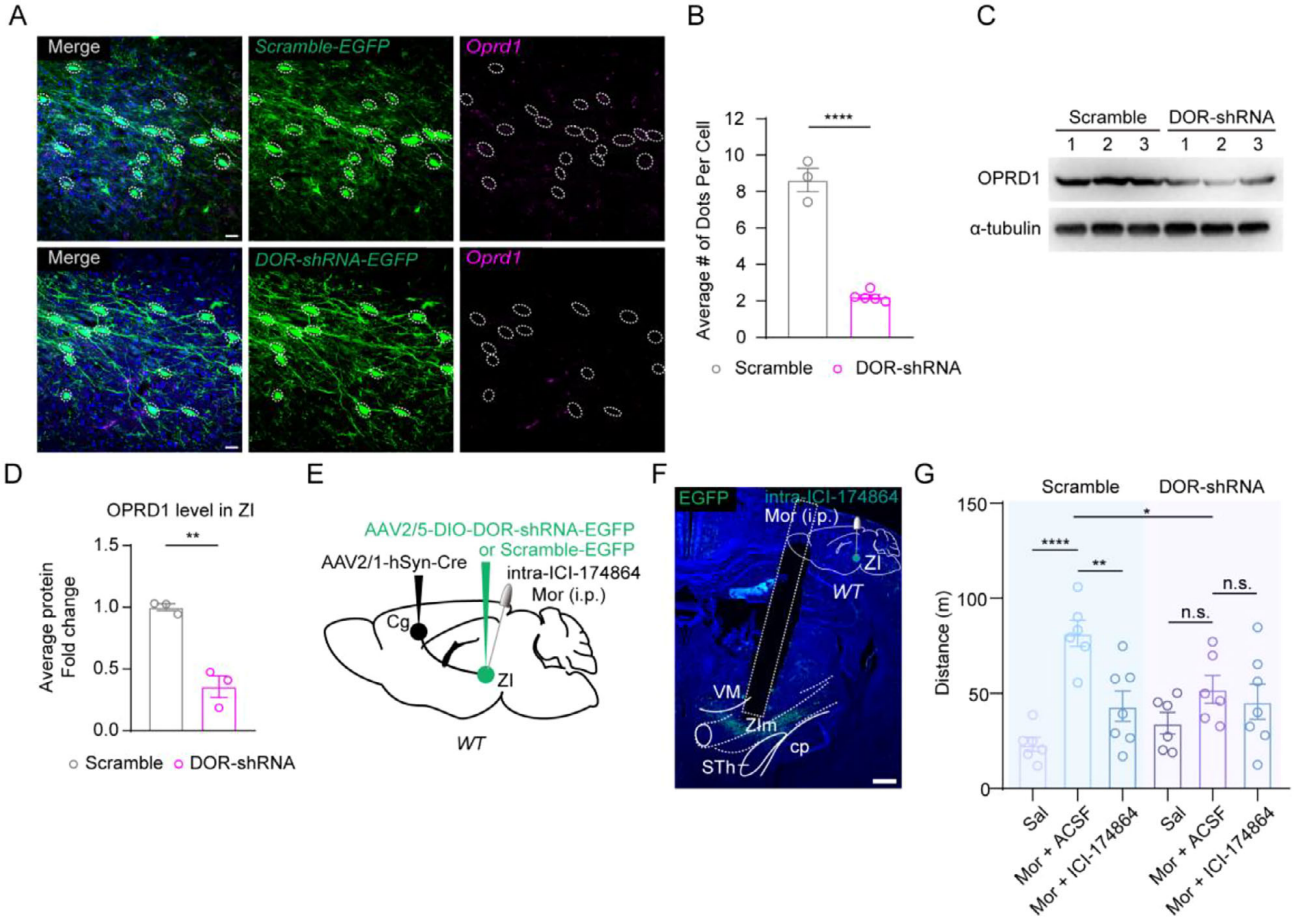
Knocking down DOR on ZIm^Cg^ effectively blocks the reduction in high‐dose morphine‐induced hyperlocomotion caused by ICI174864. A) Representative images of ZIm^Cg^ expressing Scramble‐EGFP or DOR‐shRNA‐EGFP. The expression of DOR was assessed by the specific probe. Green, EGFP; Magenta, *Oprd1*. B) Quantification of the expression of *Oprd1* in ZIm^Cg^ (t_(6)_ = 12.77, ^****^ *P* < 0.0001; two‐sided unpaired t‐test). n = 150 cells from 3 mice in Scramble group, n = 328 cells from 5 mice in DOR‐shRNA group. C, D) Western blot representation (C) and quantification (D) of OPRD1 in ZIm between groups; the blots are cropped according to their molecular weight. (t_(4)_ = 7.020, ^**^ *P* = 0.0022; two‐sided unpaired t‐test). n = 3 mice for each group. E) Schematic of virus injection. F) Schematic paradigm of drug administration and representative images of EGFP infection and cannula implantation. Scale bars, 200 µm. G) Distance travelled after i.p. injection (F_(5, 32)_ = 7.138, ^***^ *P* = 0.0001; ^****^ *P*_Sal vs. Mor_ < 0.0001, ^**^ *P*_Mor+ACSF vs. Mor+ICI174864_ = 0.0025 in Scramble group; P _Sal vs. Mor_ = 0.1871, P _Mor+ACSF vs. Mor+ICI174864_ = 0.5218 in DOR‐shRNA group; ^*^ *P*_Mor+Scramble vs. Mor+DOR‐shRNA_= 0.0256; two‐sided one‐way ANOVA with Holm‐Sidak test). n (mice) = 6 (Scramble+Sal/Mor), 6 (DOR‐shRNA+Sal/Mor), 7 (Scramble+Mor+ICI174864), 7 (DOR‐shRNA+Mor+ICI174864). ^*^ *P* < 0.05, ^**^ *P* < 0.01, ^****^ *P* < 0.0001, n.s. = no significant difference. Error bars represent s.e.m.

To confirm whether the DOR antagonist reduced the morphine‐induced hyperlocomotion through DOR expressed on the Cg‐ZIm pathway, we injected the AAV2/1‐Cre into the Cg and AAV2/5‐DIO‐DOR‐shRNA‐EGFP into the ZIm to specifically knock down DOR in ZIm^Cg^. Our RNAscope and western blot analyses demonstrated effective knockdown of DOR at both the RNA and protein levels (**Figure** **7**A–D). Subsequent behavioral assessments revealed that after DOR knockdown in ZIm^Cg^, the DOR antagonist failed to further attenuate morphine‐induced hyperlocomotion (Figure 7E–G), suggesting the pivotal role of DOR on ZIm^Cg^ in morphine‐induced hyperlocomotion.

## 3 Discussion

Drug addiction is a global issue that burdens society and public health, resulting from the pursuit of recreational drugs, and can evolve into multifaceted neurological disorders.^[^ ^63^, ^64^ ^]^ Although there have been numerous studies on the neural circuits of morphine‐induced behavioral responses,^[^ ^65^, ^66^, ^67^, ^68^, ^69^, ^70^, ^71^, ^72^ ^]^ it remains unclear whether the same pathways are necessary for morphine‐induced psychomotor effects. Here we found that Cg‐ZIm excitatory transmission was both sufficient and necessary for morphine induced hyperlocomotion, but was not involved in morphine reward or morphine withdrawal. Additionally, DOR within this circuit played a critical role in mediating the psychomotor effects of morphine. In conjunction with our prior investigations,^[^ ^3^ ^]^ this suggests the presence of separate neural pathways and complex molecular mechanisms underlying the manifestation of morphine adaptive behaviors.

Although morphine‐induced hyperlocomotion is widely used in rodents as an index of drug action on motor circuits, its association with reward learning is circuit‐specific and not always correlated. Several studies have demonstrated that hyperlocomotion and CPP are mediated by partially overlapping but dissociable circuits.^[^ ^65^, ^73^, ^74^ ^]^ For instance, D1 receptor knockout mice exhibit blunted hyperlocomotion in response to morphine but retain a normal CPP response,^[^ ^75^ ^]^ indicating that reward valuation and motor activation are separable processes. In our study, disrupting the Cg‐ZIm pathway reduced morphine‐induced hyperlocomotion without affecting CPP. This functional dissociation is consistent with previous work,^[^ ^3^ ^]^ which demonstrated that rostral VTA GABAergic projections to the dorsal raphe nucleus (DRN) regulate morphine CPP, whereas morphine's analgesic and hyperlocomotive effects remained intact. These results support a model in which opioid reward, analgesia, and motor activation are mediated by parallel but distinct neural pathways, and interventions targeting hyperlocomotion may not necessarily affect reward‐associated learning.

Various cellular types prevail in distinct regions of the ZI,^[^ ^18^ ^]^ displaying diverse patterns of connectivity within the ZI,^[^ ^18^, ^19^ ^]^ allowing it to participate in numerous physiological processes. There are inconsistent results concerning the role of ZI in locomotion.^[^ ^7^, ^14^ ^]^ Our results showed ≈50% of ZIm^Cg^ overlapped with *gad2* mRNA (Figure 1C), and selective activation of ZIm neurons downstream of the Cg but not ZIm^gad2^ neurons increased locomotion (Figure 5I; Figure S6B, Supporting Information), indicating the high heterogeneity of the ZIm and intricate microcircuitry involved in locomotion modulation. Our tracing studies further revealed that ZIm^Cg^ projects to motor‐related regions, including the motor cortex (M1/2), and striatum (Figure S14, Supporting Information),^[^ ^20^, ^76^, ^77^ ^]^ which is consistent with previous anatomical studies.^[^ ^44^, ^78^ ^]^ These converging findings support the idea that the ZIm may play an integrative role in motor control. Given these converging lines of evidence, it would be particularly fascinating to explore how the ZI—through interactions among its subdivisions, cell types, and projection targets—modulates behavior in complex sensory and motivational contexts. Future studies that dissect these microcircuits across behavioral states may offer valuable insights into the neural mechanisms of perception, decision‐making, and action selection.

Despite all opioid receptors being coupled to the Gi pathway, their effects on neural circuits can be distinct. MOR activation reduces glutamate release from the insular cortex or the thalamus to the striatum,^[^ ^79^, ^80^ ^]^ which leads to the traditional view of neural inhibition roles of morphine. In our study, we observed a morphine‐induced increase in c‐Fos expression in the Cg and enhanced activity of Cg^ZIm^ in the fiber photometry recordings. We further suggested that DOR, rather than MOR, mediates the morphine‐induced hyperlocomotion. Research has suggested that activation of DOR can enhance neural activity in the rat's insular cortex (IC) by reducing inhibitory transmission from fast‐spiking interneurons to pyramidal neurons.^[^ ^81^, ^82^ ^]^ Furthermore, studies have shown that parvalbumin (PV) neurons in the Cg express DOR, the activation of which leads to disinhibitory effects on pyramidal neurons by suppressing PV neuron activity, thereby enhancing glutamate release. Therefore, we hypothesize that morphine may exert disinhibitory effects on glutamatergic neurons by inhibiting PV interneurons via activation of DOR within Cg.

The progressive increase in locomotor activity observed during repeated morphine administration reflects behavioral sensitization (Figure 4), a well‐established hallmark of opioid‐induced neuroadaptation.^[^ ^3^, ^83^ ^]^ Chronic morphine exposure may enhance synaptic recruitment of the Cg‐ZIm pathway, consistent with prior studies showing that repeated drug administration engages cortical glutamatergic projections in mediating drug‐related behaviors.^[^ ^84^ ^]^ In parallel, elevated morphine concentrations are known to promote MOR phosphorylation, desensitization, and internalization, thereby reducing MOR signaling.^[^ ^85^ ^]^ This shift may favor engagement of alternative receptors such as DOR, which are also present within the Cg‐ZIm circuit.

Opioid receptors are distributed throughout the nervous system, reducing responses to painful stimuli and influencing reward perception.^[^ ^61^ ^]^ Activation of either the MOR or DOR has been shown to enhance locomotor activity.^[^ ^86^, ^87^ ^]^ Our results highlight the crucial role of DOR in specifically regulating morphine‐induced hyperlocomotion without affecting basal motor function. Whether the effects of DOR occurred at the pre‐ or post‐synaptic sites of the Cg‐ZIm pathway remains unclear. Results from both cannula‐based pharmacological inhibition and receptor knockout experiments suggest that postsynaptic delta‐opioid receptors (DORs) within the ZIm play a critical role in mediating morphine‐induced hyperlocomotion (Figures 6 and 7). Whether the presynaptic DOR is importantly involved in this process still awaits further investigation.

Morphine is known to have the highest affinity for MOR^[^ ^60^ ^]^ and at low concentrations, it preferentially activates MOR‐mediated G‐protein signaling without inducing robust receptor phosphorylation, desensitization, or internalization. As reviewed by Williams et al., morphine at low concentrations engages MOR without triggering the same degree of receptor regulation observed with higher‐efficacy agonists or higher doses.^[^ ^88^ ^]^ This may explain why MOR antagonism remains effective locally in the ZIm at low doses (Figure S11G, Supporting Information). Based on these findings, we propose a dose‐dependent shift in receptor and circuit engagement, whereby low‐dose morphine acts primarily via MOR within local ZIm circuits, and high‐dose morphine additionally recruits Cg‐ZIm circuits via DOR to modulate psychomotor behavior (Figures 6 and 7; Figure S11, Supporting Information). Further studies will be required to delineate the precise contributions of different opioid receptors to morphine‐induced behavioral responses across a range of concentrations.

## 4 Conclusion

In this study, we have elucidated an excitatory projection from the Cg to the ZIm that drives opioid‐induced hyperlocomotion. Inhibiting this Cg‐ZIm pathway significantly reduced both acute and chronic hyperlocomotion without impairing analgesia, while its activation mimicked morphine's motor effects. Importantly, local blockade of DOR within the ZIm suppressed hyperlocomotion, highlighting a DOR‐dependent mechanism. These findings reveal a key cortico‐thalamic circuit and receptor target underlying the psychomotor effects of morphine, offering insights for more selective opioid interventions. Overall, our study reveals key circuit and molecular contributors to high‐dose morphine‐induced hyperlocomotion, shedding light on the neurobiological basis of opioid‐driven behavioral sensitization and addiction.

## 5 Experimental Section

### Animals

All procedures were approved by the Animal Advisory Committee at Zhejiang University (ZJU201553001) and performed following the National Institutes of Health *Guidelines for the Care and Use of Laboratory Animals*. Experiments were performed on adult male C57BL/6J, Gad2‐Cre (JAX Strain: 019022), vGluT2‐Cre (JAX Strain: 016963) mice, and TAC1‐Cre (JAX Strain: 021877) mice. Four‐to‐six‐week‐old mice were used for virus injection experiments, and 8‐to‐12‐week‐old mice were used for electrophysiology and behavioral experiments. Male mice were used in behavioral tests, and both female and male mice were used in electrophysiology and immunohistochemistry experiments. Mice were socially housed until guide cannula implantation. All mice were housed at a constant temperature (22–25 °C) and humidity (40%) with a 12‐h light‐dark cycle. All behavioral tests were done during the light phase.

### Virus

For GCaMP recording, rAAV2/9‐hSyn‐FLEX‐jGCaMP7b‐WPRE‐pA (titer, 1.05 × 10^13^ gc mL^−1^) was purchased from OBiO Technology, AAV2/9‐hEF1a‐fDIO‐GCaMP6s‐WPRE‐PA (titer, 2 × 10^12^ gc mL^−1^) was purchased from Shanghai Taitool Bioscience. For retrograde tracing, AAV2/2Retro‐hSyn‐Cre‐WPRE‐pA (titer, 3 × 10^12^ gc mL^−1^) was purchased from OBiO Technology. For anterograde tracing, rAAV2/1‐hSyn‐Cre‐WPRE‐pA (titer, 1.2 × 10^12^ gc mL^−1^) and rAAV2/9‐hSyn‐DIO‐mGFP‐WPRE‐pA (titer, 5.36 × 10^12^ gc mL^−1^) were purchased from Shanghai Taitool Bioscience, scAAV2/2Retro‐hSyn‐FLEX‐Flpo‐pA (titer, 2 × 10^13^ gc mL^−1^). For tracing, rAAV‐hSyn‐DIO‐mGFP‐T2A‐Synaptophysin‐mRuby‐WPRE‐hGH polyA (titer, 8.9 × 10^12^ gc mL^−1^) was purchased from OBiO Technology. rAAV2/9‐EF1α‐DIO‐N2cG (titer, 2 × 10^12^ gc mL^−1^), rAAV2/9‐EF1G‐DIO‐EGFP‐T2A‐TVA (titer, 2 × 10^12^ gc mL^−1^), CVS‐EnvA‐AG‐ldTomato (titer, 2 × 10^9^ gc mL^−1^) were purchased from BrainCase (Shenzhen). For optogenetic experiments, rAAV2/9‐Ef1α‐DIO‐hChR2(H134R)‐mCherry‐WPRE‐pA (titer, 5.4 × 10^12^ gc mL^−1^), rAAV2/9‐mCaMKIIα‐hChR2(H134R)‐mCherry‐WPRE‐pA (titer, 4.01 × 10^12^ gc mL^−1^), rAAV2/9‐Ef1α‐DIO‐mCherry‐WPRE‐pA (titer, 2.53 × 10^12^ gc mL^−1^), rAAV2/9‐mCaMKIIα‐mCherry‐WPRE‐pA (titer, 3.82 × 10^12^ gc mL^−1^) were purchased from Shanghai Taitool Bioscience. For chemogenetic experiments, rAAV2/9‐hSyn‐DIO‐hM4Di‐mCherry‐WPRE‐pA (titer, 3.1 × 10^12^ gc mL^−1^), AAV2/9‐hEF1a‐fDIO‐hM4D(Gi)‐mCherry‐ER2‐WPRE‐pA (titer, 2.6 × 10^12^ gc mL^−1^) were purchased from Shanghai Taitool Bioscience, rAAV2/9‐hSyn‐Cre‐off‐hM4Di‐mCherry‐WPRE‐pA was purchased from OBiO Technology. For Cre‐dependent expression of shRNAs in mice, the coding sequence of shRNAs targeting mouse DOR (5'‐GTGCTATGGCCTCATGCTACT‐3') was cloned into the pAAV‐CMV‐Flex‐MIR30shRNA‐EGFP vector. AAV5‐Flex‐DOR‐shRNA‐EGFP or AAV5‐Flex‐Scramble‐shRNA‐EGFP viruses were packaged by Obio Technology (Shanghai, China). In this context, scramble refers to a randomized, non‐targeting control shRNA sequence designed to avoid specific gene silencing and minimize off‐target effects, thereby serving as a negative control to validate the specificity of the DOR‐targeting shRNA.

### Stereotaxic Surgery

Mice were anesthetized with i.p. injections of sodium pentobarbital (75 mg kg^−1^) and positioned in a stereotaxic frame (RWD, 68030, 68025, Shenzhen, China). Injections were performed with a 10‐µL syringe (Hamilton, Nevada, USA) connected to a glass micropipette with a 10–15 µm diameter tip. Syringe pumps (KD Scientific, 78‐8130, USA) were used to inject the virus at a certain speed and volume. The viruses were injected at a specific speed of 30–50 nL min^−1^, and the glass micropipette was held for 10–15 min after injection.

For recording calcium signals of postsynaptic ZIm^Cg^ neurons, AAV2/1‐hSyn‐Cre was injected into the Cg of C57BL/6 mice (150 nL per side; AP: 1.4 mm; ML: ± 0.3 mm; DV: −1.65 mm), AAV2/9‐hSyn‐FLEX‐jGCaMP7b‐WPRE‐pA was injected into the ZIm (80 nL per side; AP: −1.95 mm; ML: ± 1.4 mm; DV: −3.99 mm). For recording calcium signals of Cg^ZIm^ neurons, AAV2/2Retro‐hSyn‐Cre‐WPRE‐pA was injected into the ZIm of C57BL/6 mice (80 nL per side; AP: −1.95 mm; ML: ± 1.4 mm; DV: −3.99 mm), AAV2/9‐hSyn‐FLEX‐jGCaMP7b‐WPRE‐pA was injected into the Cg (150 nL per side; AP: 1.4 mm; ML: ± 0.3 mm; DV: −1.65 mm).

For chemogenetic inhibition of Cg^ZIm^ neurons, AAV2/2Retro‐hSyn‐Cre‐WPRE‐pA was injected into the bilateral ZIm of C57BL/6 mice (80 nL per side), rAAV2/9‐hSyn‐DIO‐hM4Di‐mCherry‐WPRE‐pA was injected into Cg (150 nL per side). For chemogenetic inhibition of postsynaptic ZIm^Cg^ neurons, AAV2/1‐hSyn‐Cre was injected into the Cg of C57BL/6 mice (150 nL per side), rAAV2/9‐hSyn‐DIO‐hM4Di‐mCherry‐WPRE‐pA was injected into the ZIm (80 nL per side).

For optogenetic activation of Cg CaMKIIα neurons, rAAV2/9‐mCaMKIIα‐mCherry‐WPRE‐pA, rAAV2/9‐mCaMKIIα‐hChR2(H134R)‐mCherry‐WPRE‐pA was injected into the Cg of C57BL/6 mice (150 nL per side). For optogenetic activation of postsynaptic ZIm^Cg^ neurons, AAV2/1‐hSyn‐Cre was injected into the Cg of C57BL/6 mice (150 nL per side), rAAV2/9‐Ef1α‐DIO‐mCherry‐WPRE‐pA, rAAV2/9‐Ef1α‐DIO‐hChR2(H134R)‐mCherry‐WPRE‐pA was injected into the ZIm (80 nL per side). For optogenetic activation of Cg^ZIm^ neurons, AAV2/2Retro‐hSyn‐Cre‐WPRE‐pA was injected into the ZIm of C57BL/6 mice (80 nL per side), rAAV2/9‐Ef1α‐DIO‐mCherry‐WPRE‐pA, rAAV2/9‐Ef1α‐DIO‐hChR2(H134R)‐mCherry‐WPRE‐pA was injected into the Cg (150 nL per side).

For optogenetic activation of ZIm Gad2 neurons, rAAV2/9‐Ef1α‐DIO‐hChR2(H134R)‐mCherry‐WPRE‐pA, rAAV2/9‐Ef1α‐DIO‐mCherry‐WPRE‐pA was injected into the ZIm of *Gad2*‐Cre mice (80 nL per side). For chemogenetic inhibition of ZIm Gad2 neurons, rAAV2/9‐hSyn‐DIO‐hM4Di‐mCherry‐WPRE‐pA was injected into the ZIm of *Gad2*‐Cre mice (80 nL per side).

For tracing the Cg‐ZIm pathway, a total volume of 80 nL containing an equal volume of rAAV2/9‐EF1α‐DIO‐N2cG and rAAV2/9‐EF1G‐DIO‐EGFP‐T2A‐TVA was injected at the ZIm (only on one side) of *Gad2*‐Cre and *Vglut2*‐Cre mice. Two weeks later, 80 nL of CVS‐EnvA‐AG‐ldTomato was injected into the ZIm (only on one side).

To retrograde label Cg^ZIm^, CTB‐555 was injected into the ZIm of 57BL/6 mice (80 nL only on one side). For anterograde tracing of Cg Vglut2 neurons to examine their axonal projections and synaptic connectivity, rAAV2/9‐hSyn‐DIO‐mGFP‐WPRE‐pA, rAAV‐hSyn‐DIO‐mGFP‐T2A‐Synaptophysin‐mRuby‐WPRE‐hGH polyA was injected into the Cg of vGluT2‐Cre mice (150 nL only on one side).

For shRNA experiments, AAV2/1‐hSyn‐Cre was injected into the Cg of C57BL/6 mice (150 nL per side; AP: 1.4 mm; ML: ± 0.3 mm; DV: ‐1.65 mm), AAV5‐Flex‐DOR‐shRNA‐EGFP or AAV5‐Flex‐Scramble‐shRNA‐EGFP was injected into the ZIm (80 nL per side; AP: −1.95 mm; ML: ± 1.4 mm; DV: −3.99 mm).

For implantation, optical fiber (200‐µm diameter; NA 0.37; Inper) and cannulas (RWD Life Science) were surgically implanted into the target brain regions. The coordinates of the ZIm implantation were as follows: AP: −1.95 mm; ML: ± 1.4 mm; DV: −3.95 mm. For Cg implantation, the coordinates were: AP: 1.4 mm; ML: ± 0.3 mm; DV: −1.60 mm. Mice were allowed 4–6 weeks for virus expression.

For in vivo pharmacological experiments, mice were allowed to recover for at least one week following cannula implantation. Prior to drug delivery, animals were habituated in the experimental environment for 30 min. Drug administration was performed via a micro‐injector connected to a 1‐µL Hamilton syringe (Hamilton, Nevada, USA) through polyethylene tubing, using an injector and guide cannula of matching length. Compounds were infused manually into the ZIm over 2–3 min, and the injector was left in place for an additional minute to allow adequate diffusion. We used 3 µm × 300 nL ICI174684, 3 µm × 300 nL β‐FNA, 1 µm × 300 nL ML 190, or 1 µg × 200 nL morphine^[^ ^89^, ^90^, ^91^, ^92^ ^]^ dissolved in ACSF for the experiments. Mice were placed in the experimental apparatus 10 min after microinjection. After completion of behavioral testing, a post‐hoc verification using CTB555 dye was performed to confirm the accuracy of the injection site and the volume spread within the ZIm.

### Fiber Photometry

To record fluorescence signals, a 488‐nm laser beam (Inper) was reflected off a dichroic mirror (MD498, Thorlabs), focused with a 10× objective lens (0.3 NA, Olympus Inc., Japan), and then coupled to an optical commutator (Inper) to record calcium signals. The commutator and implanted fiber were connected by a 1.5‐m optical fiber (200 mm O.D., 0.37 NA). The laser power at the tip of the optical fiber was adjusted to 20–40 µW to decrease laser bleaching. The GCaMP7b fluorescence was bandpass filtered (MF525‐ 39, Thorlabs). Fluorescence signals were collected using a CMOS camera. The end of the fiber was imaged at a frame rate of 1–320. Fiber photometry data were collected with Inper Studio Software (Inper) and analyzed using Inper Data Process (v0.5.9, Inper). The demodulated signal was stored using a sampling frequency of 50 Hz. First, for the time before drug injection, the signal originating from the 405‐nm excitation source was linearly regressed to the signal originating from the 470‐nm excitation source. The regression coefficients were then applied to the entire 405‐nm originating signal, scaling it to the 470‐nm originating signal. ΔF/F was then computed as (470 nm signal – fitted 405 nm signal)/fitted 405 nm signal. Finally, the ΔF/F was binned into appropriate time bins in the graphs and analyses.

### Immunohistochemistry

Immunochemistry was referred to in the previous report.^[^ ^3^ ^]^ Animals that had undergone behavioral analysis were deeply anesthetized and transcardially perfused with 0.9% saline followed by 4% paraformaldehyde (PFA) in PBS (pH 7.4). Brains were carefully removed and post‐fixed with 4% PFA for an additional 4‐6 h at 4 °C. Next, brains were transferred to 30% sucrose dissolved in PBS for 48–72 h and were then sliced into 20 or 50‐µm coronal sections using a freezing microtome (Leica, CM3050 S, Germany). The sections were stored at −20 °C in a stocking buffer (50% PBS, 30% glycol, and 20% glycerol). After washing with PBS, free‐floating sections were incubated with a blocking buffer that contained 5% goat serum and 3% bovine serum albumin (BSA) dissolved in 0.5% PBST (0.5% TritonX‐100 in PBS) for 1 h at room temperature. Sections were then incubated with primary antibodies diluted in blocking buffer overnight at 4 °C on a horizontal shaker. Primary antibodies were: anti‐cFos (1:5,000; 226008‐Synaptic System), anti‐gaba (1:500; A2052‐Sigma), anti‐glutamate (1:500; G6642‐Sigma). After incubation, the sections were rinsed four times (15 min each) with PBS and incubated with a fluorescent dye‐conjugated secondary antibody (1:400, Invitrogen or Jackson ImmunoResearch, USA) for 1–2 h at room temperature. After washing three times (15 min each) in PBST, sections were incubated with DAPI (1:1000, Invitrogen, USA) at room temperature for 5 min, washed several times, and then mounted under coverslips with Prolong anti‐fade medium (Invitrogen). The placement of the cannula was assessed based on lesions in the tissues produced by the cannula tips. The sections were imaged using a laser confocal microscope (FV3000, Olympus) or an Olympus VS120 virtual slide microscope system.

### RNAscope In Situ Hybridization

For in situ RNA hybridization, the RNAscope multiplex fluorescent reagent kit v2 assay (ACDbio) was used. Fluorescence in situ hybridization was performed according to the manufacturer's standard protocols. Coronal sections (20 µm) were prepared as described above. The following RNAscope probes (ACD Bio) were used in this study: Mm‐DsRed‐C2 (481361‐C2), RNAscope Probe‐Mm‐Camk2a‐C3 (445231‐C3), RNAscope Probe‐Mm‐Gad2 (439371), RNAscope Probe‐Mm‐Slc17a6‐C4 (319171‐C4), RNAscope Probe‐Mm‐Oprd1 (568761), RNAscope Probe‐Mm‐Oprk1 (315841), RNAscope Probe‐Mm‐Oprk1 (316111).

### Western Blotting

The mice were decapitated, and their brains were rapidly removed to ice. After performing coronal sectioning of the region where the ZI was located, the bilateral ZI tissue was captured under the dissecting microscope. Brain tissues were homogenized in RIPA lysis buffer with protease inhibitor and phosphatase inhibitor using a tissue grinder. Proteins were separated by SDS‐polyacrylamide gels and transferred to polyvinylidene difluoride membranes, followed by blocking, primary antibodies (anti‐OPRD1, 1:1000, ab176324; anti‐α‐tublin, 1:5000, ab52866), and horseradish peroxidase‐conjugated secondary antibodies incubation. The proteins were detected using enhanced chemiluminescence (ECL) regents. Quantitative analysis was performed with ImageJ software.

### Acute Brain Slice Preparation

Eight‐ to ten‐week‐old mice were deeply anesthetized and perfused with ice‐cold oxygenated (95% O_2_ and 5% CO_2_) cutting ACSF consisting of (in mM): 230 sucrose, 2.5 KCl, 10 MgSO_4_, 0.5 CaCl_2_, 1.25 NaH_2_PO_4_, 26 NaHCO_3_, 10 glucose, and 1.5 pyruvate. Coronal brain slices (250 µm for Cg and 200 µm for ZIm) were prepared using a Leica VT1000S vibratome (Leica, VT1200, Germany) in a cutting solution. Slices were first incubated in a mixture of 50% cutting solution and 50% ACSF at 32 °C for 10 min and then transferred to a mixture of 10% cutting solution and 90% ACSF to cool to room temperature for 2–3 h before recording.

### In Vitro Electrophysiology

For recordings, slices were transferred to a recording chamber perfused with recording ACSF containing (in mm): 125 NaCl, 2.5 KCl, 1.3 NaH_2_PO_4_, 25 NaHCO_3_, 2 CaCl_2_, 1.3 MgCl_2_, and 11 glucose saturated with 95% O_2_ and 5% CO_2_. Whole‐cell patch‐clamp recordings were performed at room temperature with a MultiClamp 700B amplifier (2 kHz low‐pass filtered, 10 kHz digitization, Molecular Devices, USA) and a 1440A interface (Molecular Devices, USA) with pClamp 10.4 software (Molecular Devices, USA). Fluorescent cells were visualized under a Nikon Eclipse FN1 microscope equipped with a 40 × water‐immersion lens and illuminated with a mercury lamp. Whole‐cell patch‐clamp recordings were used unless other stated. Data were collected 2 min after obtaining a stable whole‐cell configuration.

Photostimulation (473 nm, ≈2 mW, 5‐ms pulses; 589 nm, ≈2 mW, 1 s) was delivered through an optical fiber (200‐µm diameter, NA 0.22, Inper, Hangzhou, China) placed near the slice connected to a solid‐state laser. Fluorescent cells were visualized under an Eclipse FN1 microscope (Nikon) equipped with a ×40 water‐immersion lens and illuminated with a mercury lamp. To test the virus efficiency of ChR2, the current‐clamp mode was used to record the action potentials of ChR2^+^ neurons induced by blue light (473 nm, 5‐ms pulses, ≈10 mW) at 20 Hz. The baseline membrane potential was held at −45 mV. To measure the function of chemogenetic viruses, neurons expressing hM4Di‐mCherry in the Cg were recorded.

For chemogenetic inhibition, mCherry^+^ neurons were injected with 80 pA current, and the number of activated action potentials was calculated as the baseline. Next, 10 mm CNO was added to ACSF for a duration of 10 min, during which the action potentials elicited by 80 pA current injection were recorded. Finally, the CNO was washed out, and the resulting activated action potentials were recorded. The low‐chloride internal solution contained (in mmol L^−1^): 150 potassium gluconate, 5 NaCl, 10 HEPES, 1 MgCl_2_, 2 Mg‐ATP, 0.5 Na_3_‐GTP, and 0.2 EGTA (pH 7.3). Data were measured using Clampfit 10.4 and Mini Analysis software.

### Behavioral Assays

Prior to behavioral testing, mice were habituated to the testing room and handled daily for at least 3 consecutive days. For optogenetic experiments, the optical fibers were connected to a 473nm blue laser. Laser power was adjusted to 6–8 mW for ChR2 stimulation prior to each session.

### Acute Hyperlocomotion

On testing days, mice underwent room habituation for a minimum of 30 min. The psychomotor responses to morphine were assessed in an open‐field box of 45 cm (l) × 45 cm (w) × 45 cm (h). Following intraperitoneal (i.p.) injection of either saline or morphine (5, 10, or 15 mg kg^−1^), mice were immediately placed in the arena for a 10‐min session. Locomotor activity and instantaneous velocity were recorded using an overhead camera and analyzed with ANY‐maze software (version 6.35, Stoelting).

### Morphine‐Induced Locomotor, CPP, and Withdrawal

Morphine‐induced behavioral assays were conducted according to established protocols as previously described.^[^ ^3^ ^]^ A three‐chamber apparatus was used in the CPP experiments. The two conditioning chambers (20 cm × 20 cm × 50 cm each chamber) were distinguished by different wall colors (black and white) and floor patterns (bar type and holes), and separated by a corridor (10 cm × 20 cm × 50 cm). A video tracking system was used to record and analyze mouse movement.

In Figure S4H (Supporting Information), mice expressing AAV‐hM4Di‐mCherry or AAV‐tdTomato in the Cg^ZIm^ neurons were allowed to freely explore the apparatus for 15 min to assess their baseline place preference. For the subsequent conditioning sessions, mice received intraperitoneal (i.p.) injection of CNO (4 mg kg^−1^) 30 min before morphine administration (15 mg kg^−1^, i.p.) and were then confined to the white chamber for 1 h. Six hours later, the same mice were injected with saline (i.p.) and were confined to the black chamber for 1 h. The same training with saline and morphine injection was performed for two consecutive days. Twenty‐four hours after the final training session, mice were re‐exposed to the CPP chamber and allowed to explore both sides of the chamber for 15 min. Time spent in each chamber was recorded to assess morphine‐induced place preference.

In Figure 4, mice freely explored the apparatus for 15 min to record time spent in the white chamber on day 0. Six hours later, baseline locomotor activity was measured by placing mice into the white chamber for 1 h. During the conditioning session, mice were rendered morphine‐dependent via daily intraperitoneal injections of morphine in the white chamber with doses escalating from 10 to 50 mg per kg body weight for 1 h, then 6 h later, the black chamber was associated with saline injections at the same volumes for 1 h. The mice were i.p. injected with CNO 30 min before morphine administration. Locomotor activity in the white chamber was recorded daily. On Day 6 (test day), mice were allowed to freely explore the entire apparatus for 15 min. The CPP score was measured as time spent in the white chamber on day 6 subtracted by time spent in the white chamber on day 0. To assess withdrawal behaviors, immediately following the CPP test, mice were placed into their home cages and given a final dose of morphine (50 mg kg^−1^). Two hours later, the mice were injected with naloxone (5 mg kg^−1^) to induce withdrawal behaviors. Jumping and rearing number was recorded for the next 10 min in the home cage and counted offline as a physical sign of withdrawal.

### Hotplate Test

Mice were placed on a heated plate (52 °C), and the latency to the first nocifensive response (paw licking or hind‐paw withdrawal) was recorded with a 30‐s cut‐off to prevent injury. Baseline latency was measured on Day 0. Mice then received daily subcutaneous morphine (15 mg kg^−1^) for 5 days. Acute analgesic effects were assessed 40 min post‐injection on Day 1, and tolerance development was evaluated 40 min post‐injection on Day 5, using the same hot plate test procedure.

### Real‐Time Place Preference/Aversion

On day 1, mice were placed in a Plexiglas box with two connected chambers (25 cm × 25 cm × 25 cm each chamber) and allowed to freely explore for 15 min. One chamber was randomly designated as the stimulation chamber, and the other was designated as the non‐stimulation chamber. The percentage of time spent in the stimulation chamber was measured as the baseline, and mice with a bias for one side were excluded. On day 2, mice were randomly placed in either chamber and received 20‐Hz blue light pulses each time they entered the stimulation chamber until they entered the non‐stimulation chamber. Time spent in each chamber was measured for 15 min. Travel traces and time spent in each chamber were recorded by the ANY‐maze v.6.35.

### Optogenetic Stimulation Rotarod

The rotarod experiment was performed as previously described.^[^ ^17^ ^]^ Mice were trained for 3 consecutive days from 5 to 13 rpm min^−1^. Mice that were able to stay on the rotating rod at 13 rpm min^−1^ for 1 min. For the behavioral assay, mice from the same cage were placed in separate lanes on a rotating rod that was set to accelerate from 4 to 40 rpm min^−1^ in 5 min. The test procedure was repeated for 6 trials separated by 15‐min inter‐trial intervals. In the optogenetic stimulation test, 6 epochs alternated between light off and light on periods, beginning with the light off epoch. Blue light (473 nm, ≈8 mW, 5‐ms pulses) was applied continuously until the mice fell from the rotating rod during light‐on periods. Lasers at wavelengths of 473 nm (blue) were applied and controlled with an optogenetic system (Inper, Aurora‐200, Hangzhou, China) at 8 mW for blue illumination (473 nm, 20 Hz, 5 ms pulse width) in ChR2 and mCherry‐expressing mice.

### Marble Burying Test

The marble burying test was conducted as previously described.^[^ ^93^ ^]^ Individual food‐restricted mice were placed in standard empty cages containing ≈7.5 cm of clean standard bedding, and 20 black marbles were positioned in 5 grid patterns throughout the cage. Mice were allowed to explore the cage freely for 30 min, and then the number of successfully buried marbles was counted. A marble was defined as buried when 25% of it was visible. The experiment was recorded with a digital video camera. In between subjects, a new cage containing fresh bedding was used, and marbles were cleaned with 15% isopropyl alcohol diluted in ddH_2_O.

### Optogenetic Stimulation Open Field Test

Mice were placed in an open field arena, one was a rectangular open field (50 cm × 50 cm × 50 cm), and the other was a pentagram open field (side length 30 cm, height 30 cm), then the mice were allowed to freely explore for 9 min. A 20‐Hz photostimulation was delivered during the 4th–6th min. Time spent in the center zone was assayed as a measure of anxiety‐like behavior. Locomotor activity was evaluated as the total distance traveled that was recorded automatically by the ANY‐maze v.6.35.

### Optogenetic Stimulation Elevated Plus Maze Test

The maze consisted of four crossing arms (two open and two closed) placed at a height of 50 cm above the ground. Mice were placed in the center of the platform at the beginning of the experiment and allowed to freely explore the maze for 9 min. A 20‐Hz, 5‐ms photostimulation was delivered during the 4th–6th min. Locations of mice were tracked automatically by the ANY‐maze v.6.35.

### Chemogenetic Manipulation

For chemogenetic inhibition, the designer drug CNO (4 mg kg^−1^, i.p.; Sigma–Aldrich, St Louis, MO) was administered 30 min before the behavior test or morphine administration.

### Quantification and Statistical Analysis

One‐way ANOVA, two‐way ANOVA, or *t*‐tests were used to analyze data when applicable. Non‐parametric tests were used if the data did not meet the assumptions of the intended parametric test. All of the statistical details of experiments can be found in the figure legends. Data are presented as means ± standard errors of the means (s.e.m.). Significance was defined as *P* < 0.05. All data were collected randomly. Sample sizes were chosen based on those used in previous papers. Statistical analyses were performed with GraphPad Prism 8.0 or Origin 8 software.

## Conflict of Interest

The authors declare no conflict of interest.

## Author Contributions

All authors participated in the collection and discussion of the data and revision of the manuscript. C.‐Y.L., X.‐M.L. initiated and designed the research and wrote the manuscript. C.‐Y.L. performed all experiments and analyzed the results. X.‐F.S., K.‐L.C., D.Z., Y.Z., S.‐Z.X, X.‐D.Y. helped collect and interpret the results. J.‐D.C. and J.S. contributed to the discussion of the results. H.‐Q.H., Y.L., M.Y., and X.‐M.L. supervised the entire project.

## Supporting information



Supporting Information

## Data Availability

The data that support the findings of this study are available from the corresponding author upon reasonable request.
