# Supplementary material for: Delta Opioid Receptors within the Cortico‐Thalamic Circuitry Underlie Hyperactivity Induced by High‐Dose Morphine
Source: Adv Sci (Weinh). 2025 Nov 30;13(9):e03831. doi: 10.1002/advs.202503831 (PMC12904023; doi:10.1002/advs.202503831)
Supplement: Supplementary file 1 — Supporting Information [file ADVS-13-e03831-s001.docx]

Supporting Information

Delta Opioid Receptors within the Cortico-thalamic Circuitry Underlie Hyperactivity induced by High-dose Morphine

*Chun-Yue Li, Huiqian Huang*, Xiao-Fan Shen, Ke-Lei Cao, Di Zheng, Yi Zhu, Shi-Ze Xie, Xiao-Dan Yu, Hao Wang, Jia-Dong Chen, Jie Shi, Yue Li*, Min Yan*, Xiao-Ming Li**

**This PDF file includes:**

Figures S1 to S14

**Other Supporting Information for this manuscript includes the following:**

Table S1.


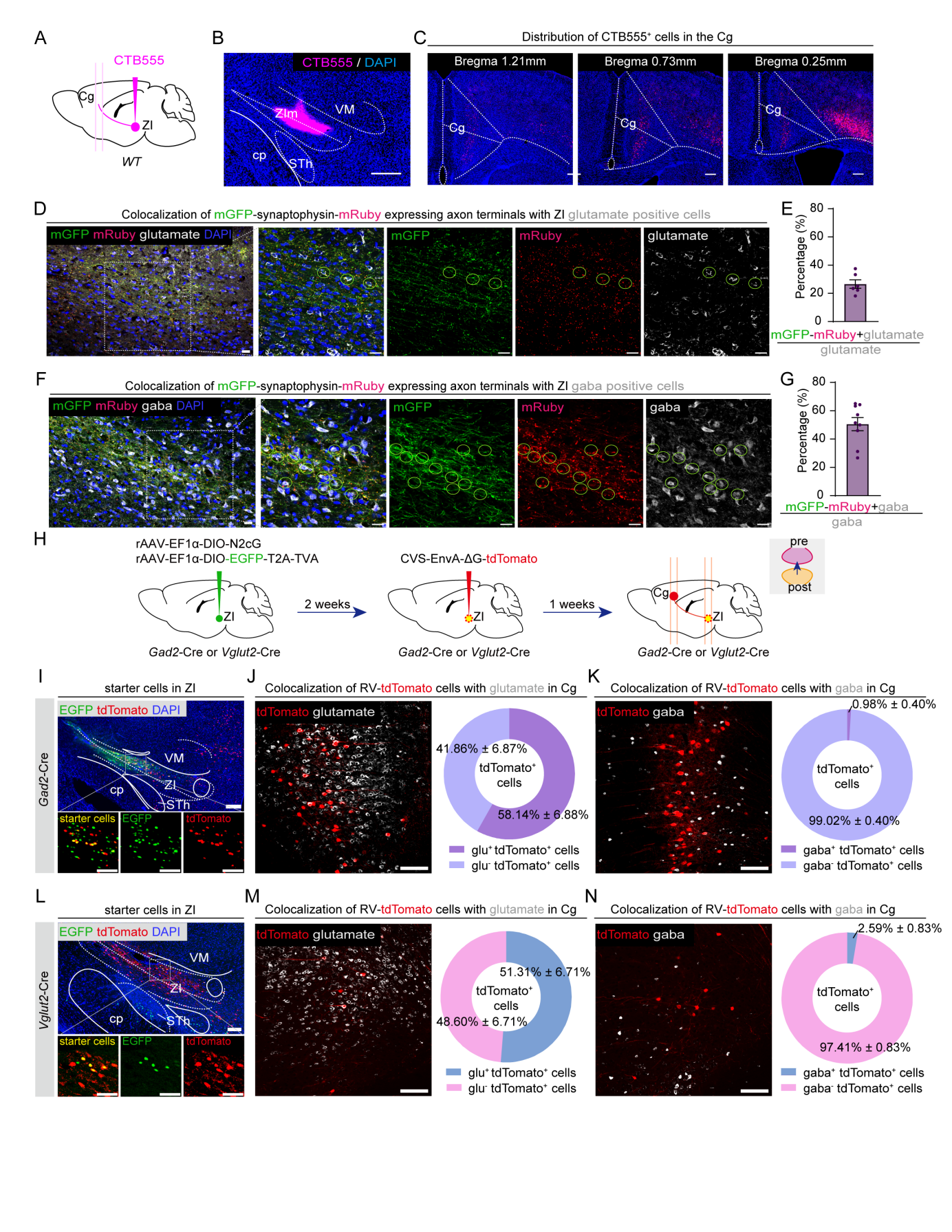


**Figure S1.** Cg v*glut2*^+^ neurons synapse onto ZIm neurons. (A, B) Scheme for infection of ZI neurons with CTB555. Scale bar, 50 μm. (C) Represages show retrograde labeling with CTB555 in the Cg, ranging from Bregma + 1.21 to + 0.25 mm. Scale bars, 200 μm. (D, F) mGFP-Synaptophysin-mRuby axon terminals in the ZIm of *Vglut2*-Cre mice. High-maentative imgnification micrographs showing immunostaining of mGFP-Synaptophysin-mRuby vs. glutamate (D) or vs. gaba (F) in ZIm. (E, G) Quantitative analyses indicating Cg *Vglut2* neurons mostly innervated ZIm gaba neurons (*n* = 3 mice). Scale bars, 20 μm. (H) Experimental design for retrograde transsynaptic viral injection. (I, L) Starter cells co-expressing TVA-GFP (green) and tdTomato (red) in the ZIm of G*ad2*-Cre (I) and *Vglut2*-Cre mice (L). (J, K, M, N, left panel) Input cells (tdTomato) to ZIm gad2 (J, K) and vglut2 neurons (M, N) mostly colocalized with glutamate (J, M, left panel), but not gaba (K, N, left panel). (J, K, M, N, right panel) Pie charts represent the fraction of different cell types projecting from the Cg to ZIm gad2 (J, M, right panel) and vglut2 neurons (K, N, right panel). (*n* = 3 mice for each group). Scale bars, (I, L, upper panel, and J, K, M, N, left panel) 100 μm, (I, L, lower panel) 50 μm. *** P < 0.001. Error bars represent s.e.m.

**Figure S2. CTB-based retrograde tracing identifies Cg inputs to zona incerta, intermediate rostrocaudal division (ZIm).** (A) Schematic of double retrograde labeling and overlay of CTB cells in ZI. (B) Example coronal sections showing injection of CTB488 and CTB555 into the medial ZI (ML: +0.9 mm) and lateral ZI (ML: +1.75 mm), respectively. (C) Confocal images showing the retrogradely labeled neurons in Cg. (D) Quantitative analyses of the distribution of Cg neurons projecting to the medial ZI (ML: +0.9 mm) and lateral ZI (ML: +1.75 mm). (E) Schematic of double retrograde labeling and overlay of CTB cells in ZI. (F) Example coronal sections showing injection of CTB488 and CTB555 into the medial ZI (ML: +0.9 mm) and rostral ZI, respectively. (G) Confocal images showing the retrogradely labeled neurons in Cg. (H) Quantitative analyses of the distribution of Cg neurons projecting to the medial ZI (ML: +0.9 mm) and rostral ZI. (I) Schematic of double retrograde labeling and overlay of CTB cells in ZI. (J) Example coronal sections showing injection of CTB488 and CTB555 into the medial ZI (ML: +0.9 mm) and caudal ZI, respectively. (K) Confocal images showing the retrogradely labeled neurons in Cg. (L) Quantitative analyses of the distribution of Cg neurons projecting to the medial ZI (ML: +0.9 mm) and caudal ZI. High-magnification images show CTB cells in boxed regions (C,G,K). Data are shown as the mean ± SEM. Each individual dot in the bar charts represents data from a single mouse. Scale bars, 100 μm.


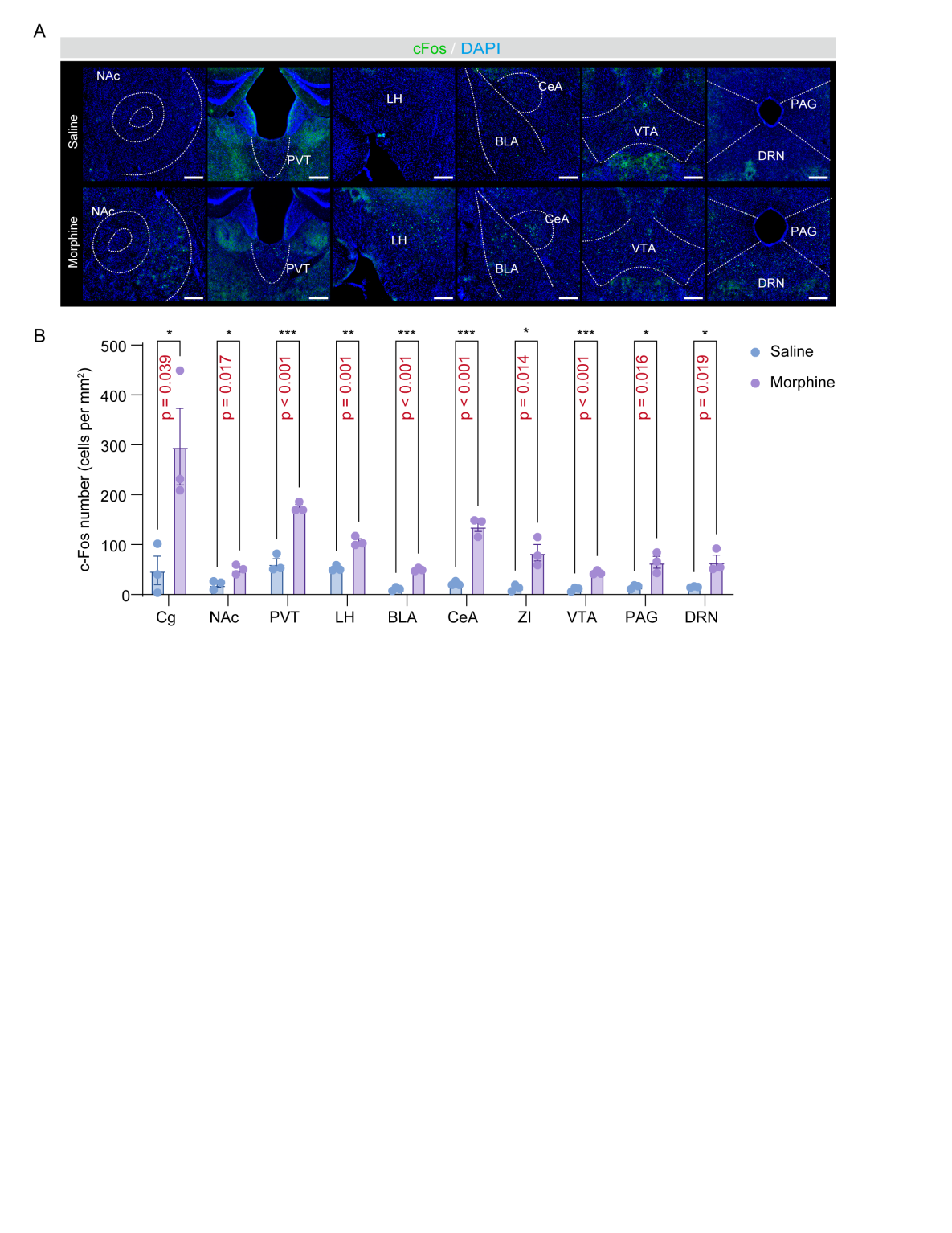


**Figure S3.** Brain area–specific activation by systemic morphine administration in mice. (A) Representative images of c-Fos–immunostained brain sections of the Cg, NAc, PVT, LH, BLA, CeA, ZIm, VTA, PAG and DRN. Scale bar: 100 μm. (B) Quantification of the density of c-Fos positive cells in each brain region. *p* values are presented in the figure. (n = 3 for each group; two-sided unpaired t test with Holm-Sidak multiple comparisons). Data are shown as the mean ± SEM. *P < 0.05, **P < 0.01, ***P < 0.001.

**
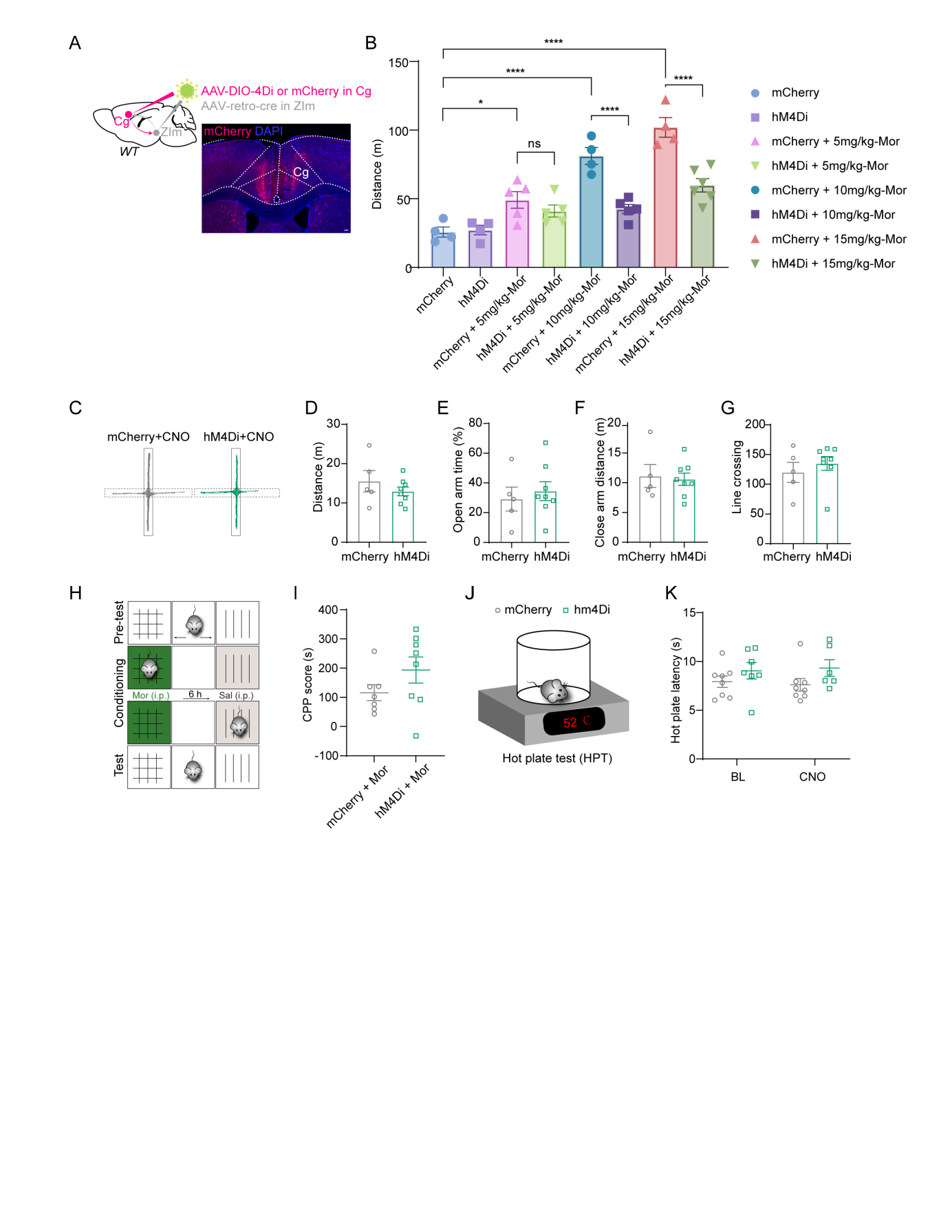
**

**Figure S4.** Chemogenetic inhibition Cg-ZIm pathway does not impact anxiety-like behaviors, morphine reward, and analgesia. (A) Experimental and schematic diagram showing injection of AAV-retro-cre and AAV2/9-DIO-hM4Di-mCherry into the ZIm and Cg of WT mice. Scale bars, 100 μm. (B) Distance traveled (F_(7, 29)_ = 24.17, **** P < 0.0001; ** P _mCherry+Sal vs. mCherry+5mg/kg-Mor_ = 0.0098; **** P _mCherry+Sal vs. mCherry+10mg/kg-Mor_ < 0.0001; **** P _mCherry+Sal vs. mCherry+15mg/kg-Mor_ < 0.0001; P _mCherry+5mg/kg-Mor vs. hM4Di+5mg/kg-Mor_ = 0.4376; **** P _mCherry+10mg/kg-Mor vs. hM4Di+10mg/kg-Mor_ < 0.0001; **** P _mCherry+15mg/kg-Mor vs. hM4Di+15mg/kg-Mor_ < 0.0001, two-sided one-way ANOVA with Holm-Sidak test) after i.p. injections. n (mice) = 4 (mCherry + Sal), 4 (hM4Di + Sal), 5 (mCherry + 5mg/kg-Mor), 5 (hM4Di + 5mg/kg-Mor), 4 (mCherry + 10mg/kg-Mor), 5 (hM4Di + 10mg/kg-Mor), 4 (mCherry + 15mg/kg-Mor), and 6 (hM4Di + 15mg/kg-Mor). (C-G) Representative elevated plus maze tracks (C) and quantification (D, t_(11)_ = 1.011, P = 0.3339; E, t_(11)_ = 0.523, P = 0.6113; F, t_(11)_ = 0.2783, P = 0.786; G, t_(11)_ = 0.759, P = 0.4638; two-sided unpaired t-test) show no significant impact of chemoinhibition on animals’ anxiety-like behavior in both hM4Di- (n = 8) and mCherry-expressing control mice (n = 5). (H) Experimental schematics of the conditioned place preference (CPP) assay. (I) CPP scores for mice following chemoinhibition of the Cg-ZIm circuit during morphine treatment (t_(13)_ = 1.423, P = 0.1783; two-sided unpaired t-test). n (mice) = 7 (mCherry + Mor), 8 (hM4Di + Mor). (J, K) Paw withdrawal latency was assessed by the hot plate test (F_(1, 4)_ = 0.2215, P = 0.6624; two-sided two-way ANOVA with Sidak test). n (mice) = 8 (mCherry), 7 (hM4Di). n.s. = no significant difference. Data are shown as the mean ± SEM. *P < 0.05, ****P < 0.0001, n.s. = no significant difference.

**
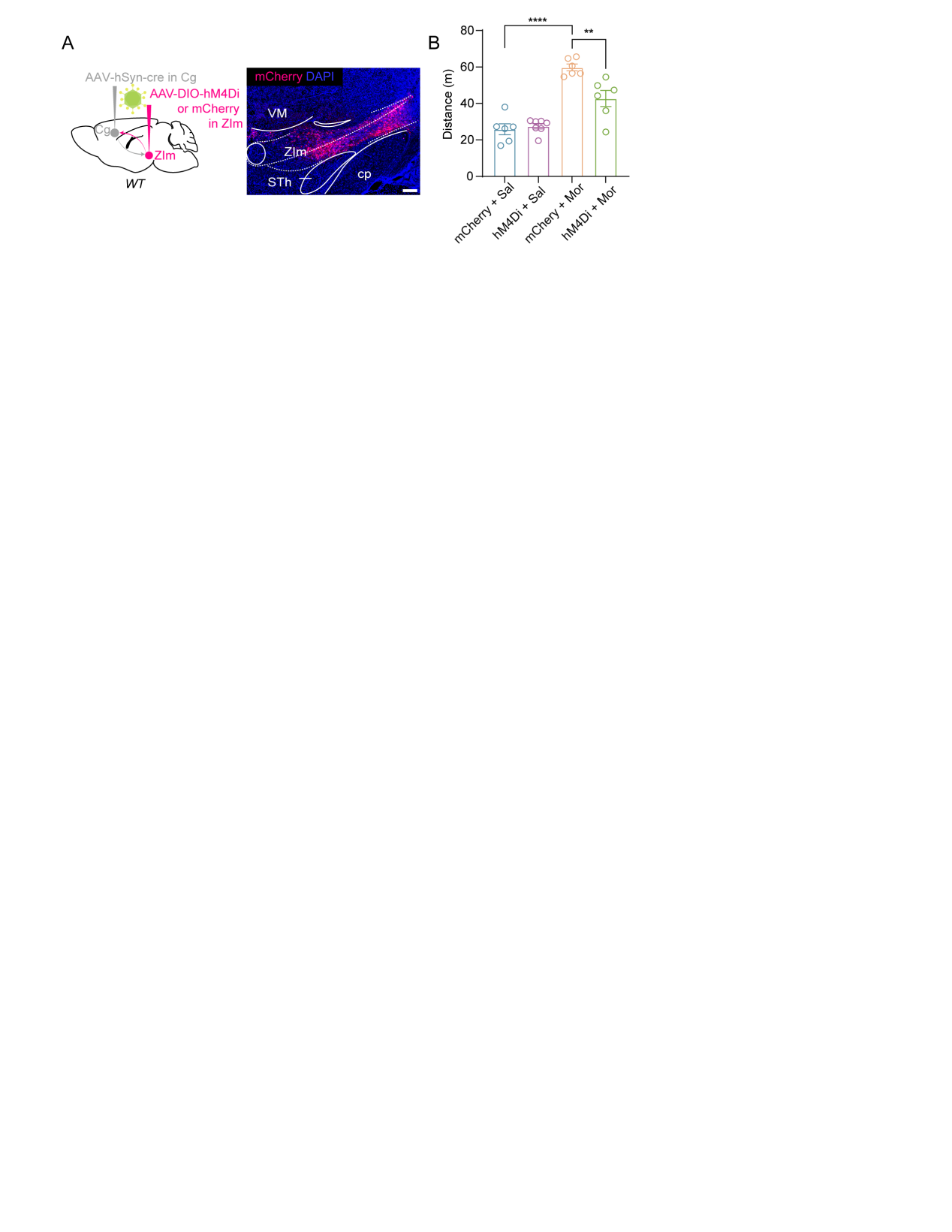
**

**Figure S5.** Chemogenetic inhibition of ZIm^Cg^ postsynaptic neurons decreased the morphine-induced hyperlocomotion. (A) Experimental and schematic diagram showing injection of AAV-hSyn-Cre and AAV-DIO-hM4Di-mCherry into the Cg and ZIm of WT mice. Scale bars, 100 μm. (B) Distance traveled after i.p. injections (F_(3, 21)_ = 30.38, **** P < 0.0001, **** P_mCherry+Sal vs. mCherry+Mor_ < 0.0001; ** P_mCherry+Mor vs. hM4Di+Mor_ = 0.0014; two-sided one-way ANOVA with Sidak test). n (mice) = 6 (mCherry + Sal), 7 (hM4Di + Sal), 6 (mCherry + Mor), and 6 (hM4Di + Mor). ** P < 0.01, **** P < 0.0001. Error bars represent s.e.m.


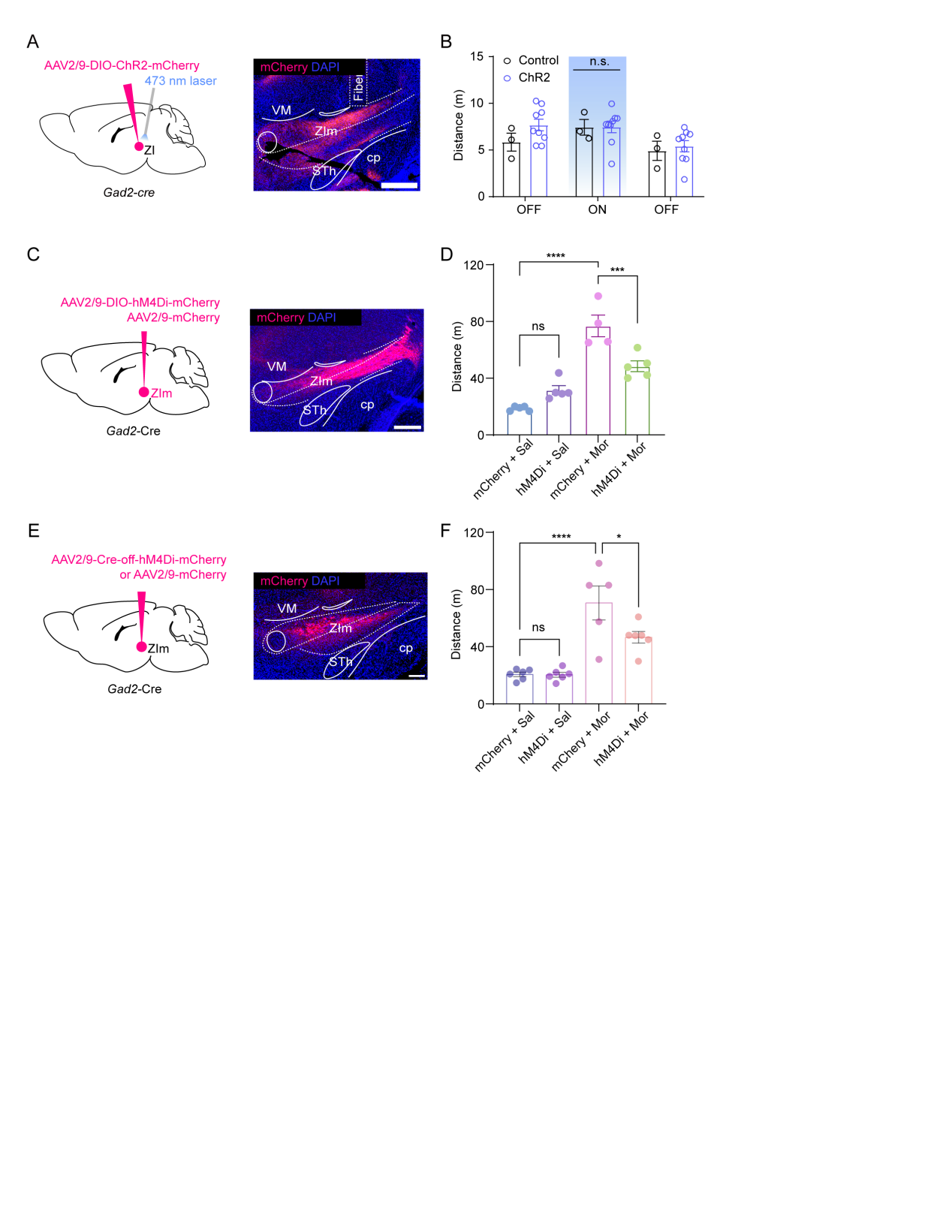


**Figure S6.** Role of the ZIm neurons in morphine-induced hyperlocomotion**.** (A) Schematic diagram showing injection of AAV2/9-DIO-ChR2-mCherry into the Cg of *Gad2*-Cre mice, followed by optical fiber implantation above the ZIm. (B) Quantitative analyses of total travel distance (F_(2, 20)_ = 0.9700, P = 0.3962; P > 0.9999 for laser ON stage comparison; two-sided two-way ANOVA with Sidak test) before, during, and after photostimulation gad2-ChR2^+^ ZIm neurons in the open field test. n (mice) = 3 (Control) and 8 (ChR2). (C) Experimental and schematic diagram showing injection of AAV2/9-DIO-hM4Di-mCherry into the ZIm of *Gad2*-Cre mice. Scale bars, 200 μm. (D) Distance traveled (F_(3, 15)_ = 34.88, **** P < 0.0001; **** P _mCherry+Sal vs. mCherry+Mor_ < 0.0001; *** P _mCherry+Mor vs. hM4Di+Mor_ = 0.0008; two-sided one-way ANOVA with Sidak test) after i.p. injections. n (mice) = 5 (mCherry + Sal), 5 (hM4Di + Sal), 4 (mCherry + Mor), and 5 (hM4Di + Mor). (E) schematic of Cre-off strategies used to express hM4Di in ZIm^non-gad2^ neurons. Scale bars, 100 μm. (F) Distance traveled after i.p. injections (F_(3, 19)_ = 17.30, **** P < 0.0001, **** P_mCherry+Sal vs. mCherry+Mor_ < 0.0001; * P_mCherry+Mor vs. hM4Di+Mor_ = 0.0166; * P _hM4Di+Sal vs. hM4Di+Mor_ = 0.0129; two-sided two-way ANOVA with Sidak test). n (mice) = 6 (mCherry + Sal), 5 (mCherry + Mor), 6 (hM4Di + Sal), and 6 (hM4Di + Mor). *P < 0.05, ***P < 0.01, ****P < 0.0001, n.s. = no significant difference. Error bars represent s.e.m.


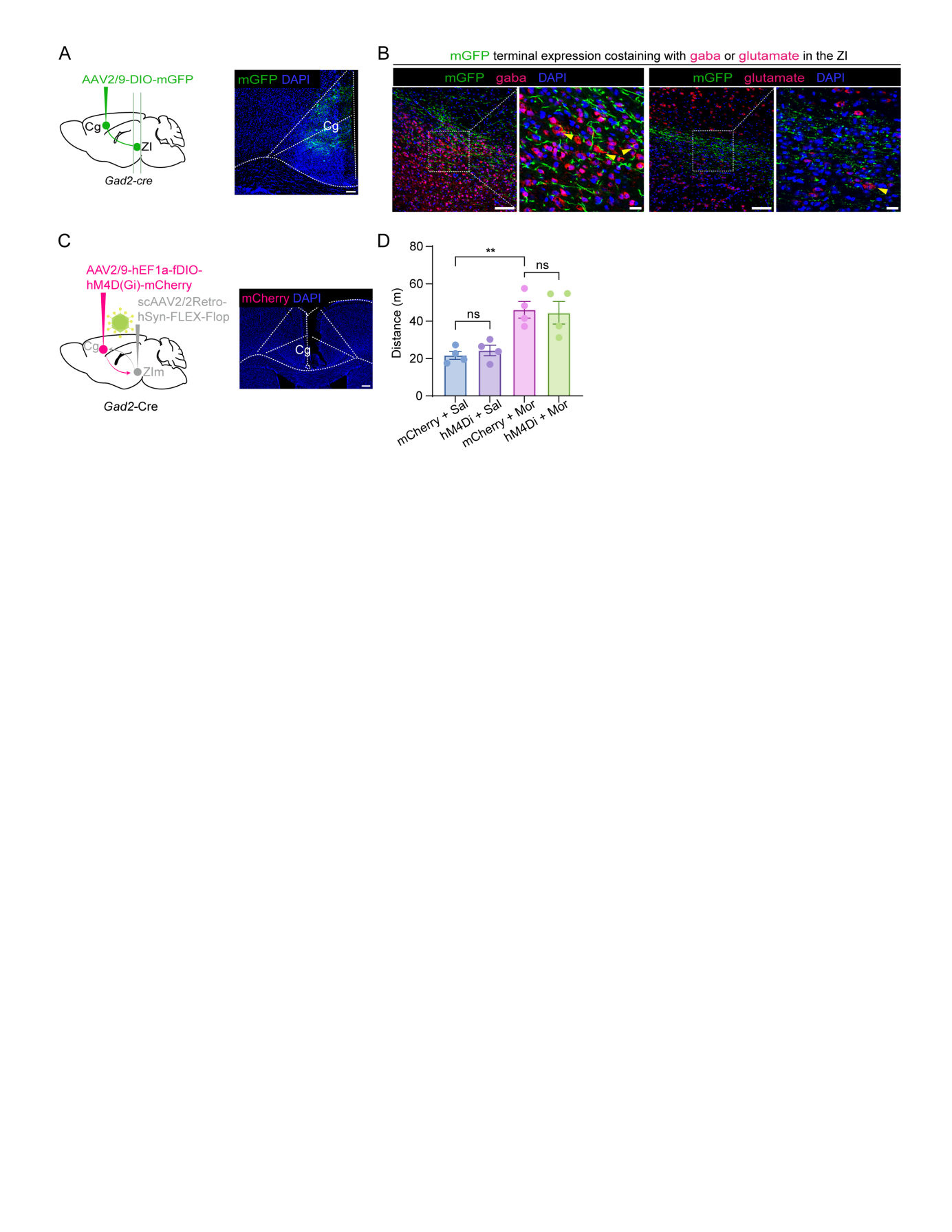


**Figure S7. Chemogenetic inhibition of GABAergic Cg neurons projecting to the ZIm.** (A) Schematic diagram and example coronal brain sections showing injections of AAV2/9-DIO-mGFP in the Cg of *Gad2*-Cre mice. (B) Representative images showing mGFP terminal expression costaining with gaba (red) or glutamate (red) in the ZIm. Scale bars, 100 and 20 μm. (C) Experimental and schematic diagram showing injection of Retro-DIO-Flp into the ZI and FDIO-4Di into the Cg of *Gad2*-Cre mice. (D) Distance traveled (F_(3, 12)_ = 9.769, ** P = 0.0015; P _mCherry+Mor vs. hM4Di-Mor_ = 0.8876; ** P _mCherry+Sal vs. mCherry+Mor =_ 0.0039; two-sided one-way ANOVA with Holm-Sidak test) after i.p. injections. n (mice) = 4 (mCherry + Sal), 4 (hM4Di + Sal), 4 (mCherry + Mor), and 4 (hM4Di + Mor). Data are shown as the mean ± SEM. **P < 0.01, n.s. = no significant difference. Scale bars (A, C), 100 μm.


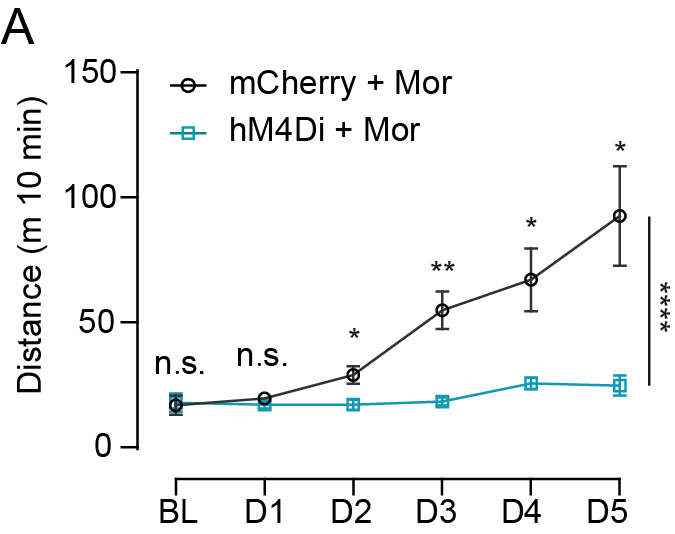


**Figure S8 related to Figure 4.** (A) Total distance traveled in response to morphine administration (10 mg/kg) while chronically reducing Cg-ZIm circuit activity during the initial 10 min (F_(5, 120)_ = 10.93, **** P < 0.0001; two-sided two-way ANOVA with Sidak test). *P < 0.05, **P < 0.01, ****P < 0.0001, n.s. = no significant difference. Error bars represent s.e.m.


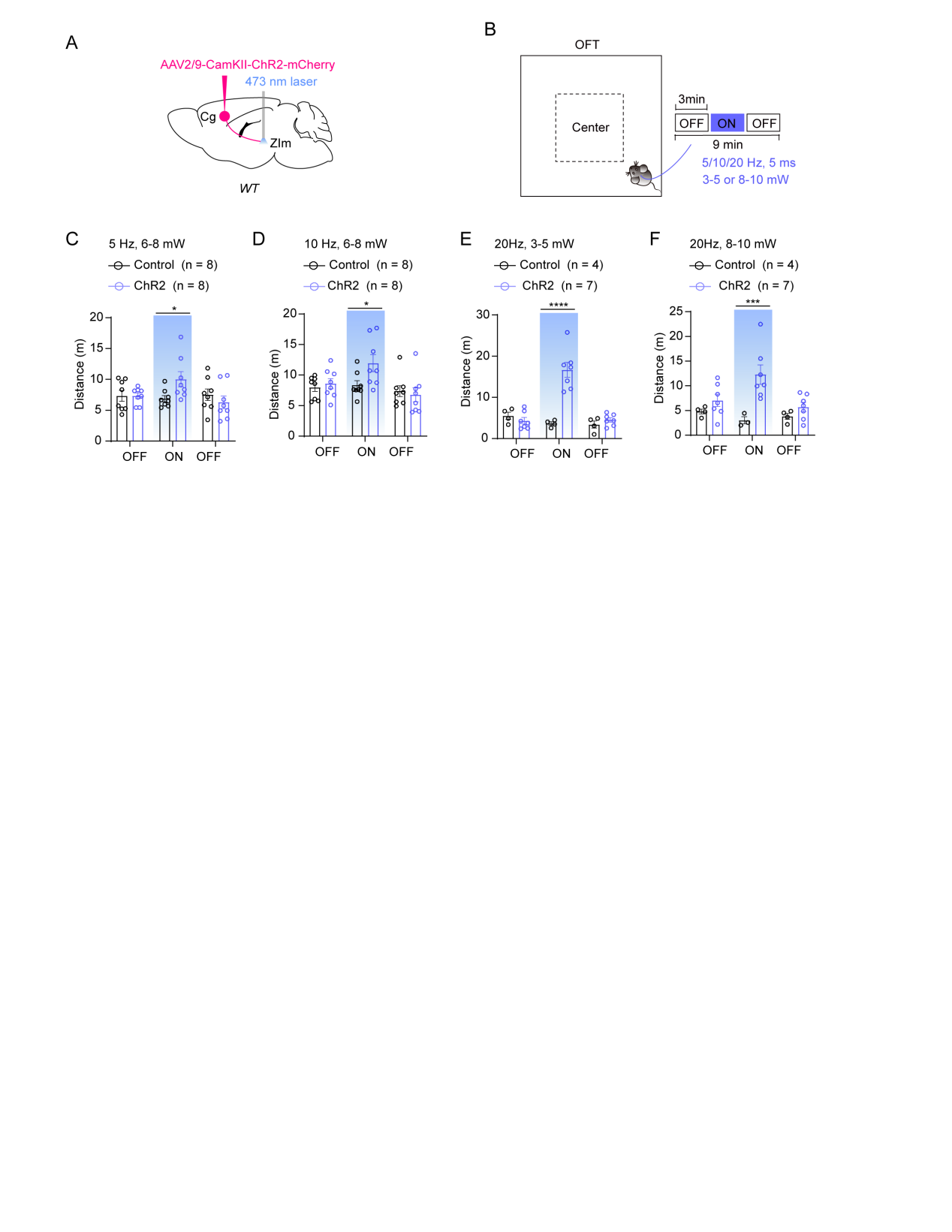


**Figure S9.** Activation of the Cg-ZIm circuit by varying light frequencies and intensities augments general locomotion in mice. (A) Schematic diagram showing injection of AAV2/9-CaMKⅡ-ChR2-mCherry and AAV2/9-CaMKⅡ-mCherry into the Cg of WT mice, followed by optical fiber implantation above the ZIm. (B) Experimental schematics of the open field test. (C-F) Total travel distance of mice with photostimulation of Cg-ZIm pathway with different stimulation frequency (5 Hz, distance, F_(2, 28)_ = 5.331, * P = 0.0109; * P = 0.0462 for laser ON stage comparison; 10 Hz, distance, F_(2, 28)_ = 3.621, ** P = 0.0399; * P = 0.0357 for laser ON stage comparison), laser power (3-5 mW, distance, F_(2, 18)_ = 17.36, **** P < 0.0001; **** P < 0.0001 for laser ON stage comparison; 8-10 mW, distance, F_(2, 17)_ = 6.509, ** P = 0.008; *** P = 0.0008 for laser ON stage comparison). * P < 0.05, *** P < 0.001, **** P < 0.0001, n.s. = no significant difference. Error bars represent s.e.m. For all figures: two-sided two-way ANOVA with Sidak test.


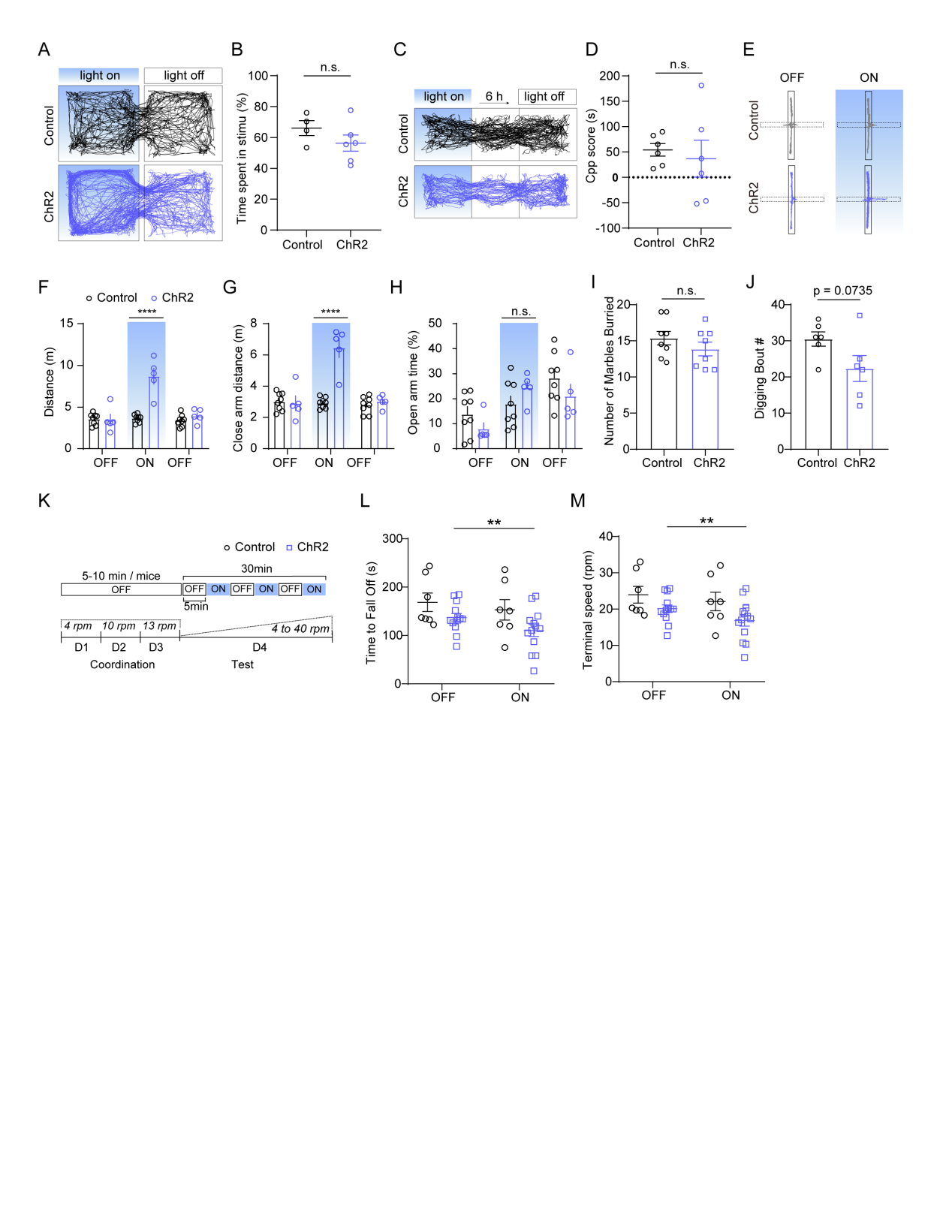


**Figure S10.** Associated behavioral side effects of Cg-ZIm circuit activation. (A) Representative tracks. (B) Quantification shows no significant impact of light stimulation on animals’ place preference in both ChR2 and control mice (t_(8)_ = 1.286, P = 0.2343). (C) Representative tracks. (D) Quantification shows no significant impact of light stimulation paired on animals’ place preference between ChR2 and control mice (t_(10)_ = 0.4493, P = 0.6628). (E-H) Representative tracks (E) and quantification (F-H) analyses of total travel distance (F_(2, 22)_ = 20.26, **** P < 0.0001; **** P < 0.0001 for laser ON stage comparison), close arm distance (F_(2, 22)_ = 21.81, **** P < 0.0001; **** P < 0.0001 for laser ON stage comparison), and open arm time (F_(2, 22)_ = 4.618, P = 0.0212; P = 0.4898 for laser ON stage comparison) before, during, and after photostimulation Cg-ZIm circuit in the EPM. (I) Almost equal buried marbles were counted between ChR2 and control mice (t_(14)_ = 1.118, P = 0.2823). (J) Digging behavior has a slight tendency to decrease in ChR2 compared with control mice (t_(10)_ = 1.999, P = 0.0735). (K) Experimental schematics. (L, M) Statistical analysis of the time of fall off (L, F_(1, 18)_ = 0.7048, P = 0.4122; P = 0.0873 for laser ON stage comparison), and terminal speed (M, F_(1, 18)_ = 0.8014, P = 0.3825; P = 0.0819 for laser ON stage comparison) upon light stimulation of Cg-ZIm circuit. ** P < 0.01, **** P < 0.0001, n.s. = no significant difference. Error bars represent s.e.m. For F, G, H, L, M: two-sided two-way ANOVA with Sidak test. For B, D, I, J: two-sided unpaired t-test.

**
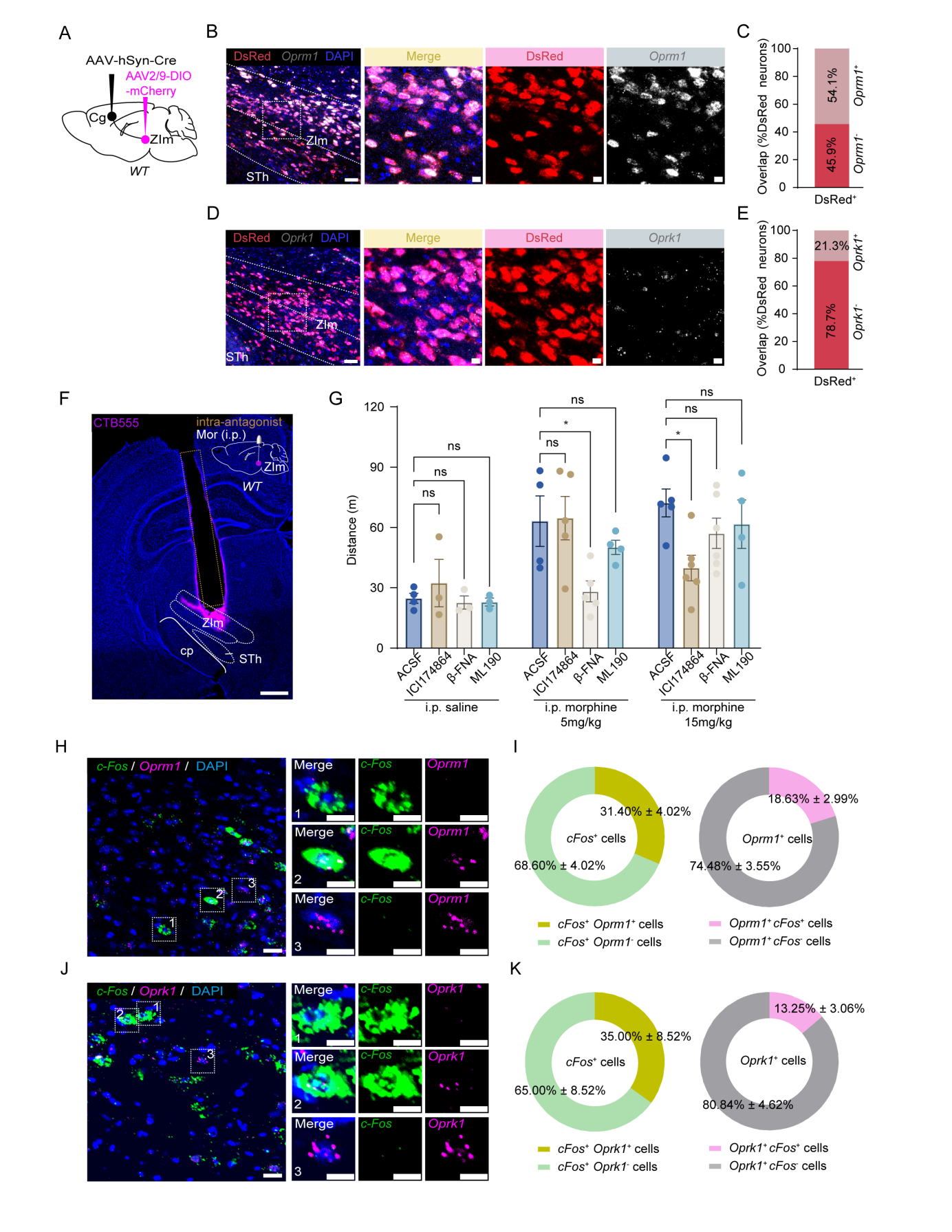
**

**Figure S11.** MOR, KOR expression, and the effects on morphine-induced hyperlocomotion. (A) Schematic. (B) *Left*: a representative image of ZIm showing DsRed-labeled ZIm that were immunopositive for *Oprm1*. Scale bars, 100 μm. *Right*: magnified image shows the boxed area; scale bar, 10 μm. (C) Quantification of co-localization between DsRed- and *Oprm1*-positive neurons in ZIm (n = 3 mice). (DsRed ^+^ *Oprm1*^+^ / DsRed ^+^, 88.95% ± 8.689%; DsRed^+^ *Oprm1*^-^/ DsRed ^+^, 76.55% ± 8.622%). (D) *Left*: a representative image of ZIm showing ChR2-DsRed-labeled ZIm that were immunopositive for *Oprk1*. Scale bars, 100 μm. *Right*: magnified image shows the boxed area; scale bar, 10 μm. (E) Quantification of co-localization between DsRed- and *Oprk1*-positive neurons in ZIm (n = 3 mice). (DsRed ^+^ *Oprk1*^+^ / DsRed ^+^, 32.89% ± 2.531%; DsRed^+^ *Oprk1*^-^/ DsRed ^+^, 122.4% ± 5.692%). (F) Schematic paradigm of drug administration and representative images of CTB555 infection and cannula implantation. Scale bars, 100 μm. (G) Statistics of locomotor distance for saline or morphine without opioid receptors antagonist and with antagonist (F_(11, 540)_ = 4.882, **** P < 0.0001; * P_ACSF+Mor vs. β-FNA+Mor_ = 0.0305; * P_ACSF+Mor vs. ICI174864+Mor_ = 0.0280; two-sided one-way ANOVA with Sidak test). In the saline group, the number of mice receiving ACSF, ICI-174864, β-FNA, and ML 190 injections was 4, 3, 3, and 3, respectively; for the 5 mg/kg morphine group, the numbers were 4/5/5/4; and for the 15 mg/kg morphine group, 4/6/6/4. (H-K) The proportion of *Oprm1*^+^ and *Oprk1*^+^ neurons expressing c-Fos following morphine administration. (H) Representative images for morphine-induced c-Fos expression co-staining with opioid receptors *Oprm1* in ZIm. Scale bars, 20 μm. Insets, high-magnification micrographs showing c-Fos-expression only (1), co-expression (2), and *Oprm1*-expression only (3) neurons. Scale bars, 10 μm. (I) Quantitative analyses for the percentage of *Oprm1* or *c-Fos* expressed neurons in ZIm. (J) Representative images for morphine-induced c-Fos expression co-staining with opioid receptors *Oprk1* in ZIm. Scale bars, 20 μm. Insets, high-magnification micrographs showing c-Fos-expression only (1), co-expression (2), and *Oprk1*-expression only (3) neurons. Scale bars, 10 μm. (K) Quantitative analyses for the percentage of *Oprk1* or *c-Fos* expressed neurons in ZIm. Results are from n = 3 mice. Error bars represent s.e.m. * P < 0.05, n.s. = no significant difference. Error bars represent s.e.m. For all figures: two-sided one-way ANOVA with Sidak test.


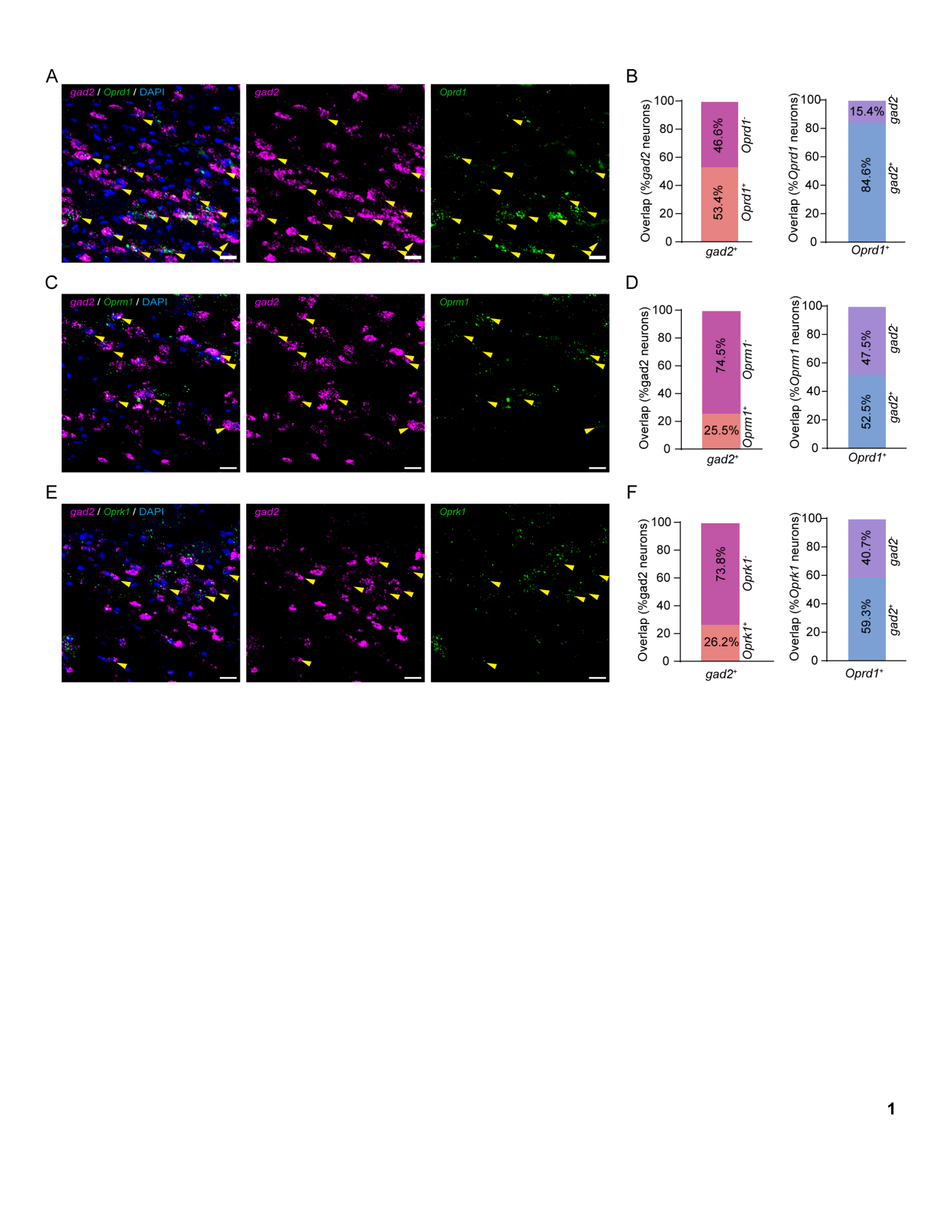


**Figure S12. Opioid receptors are expressed in either gad-positive or gad-negative neurons within ZIm.** (A) Typical images of *gad2*, and *oprd1* expression in the ZIm. (B) Quantitative analyses for the percentage of *Oprd1* or *gad2* expressed neurons in ZIm. (*left*: *gad2*^+^ *Oprd1*^+^ / *gad2*^+^, 53.36% ± 2.50%; *gad2*^+^ *Oprd1*^-^/ *gad2*^+^, 46.64% ± 2.50%; *right*: *Oprd1*^+^ *gad2*^+^ / *Oprd1*^+^, 84.63% ± 1.57%; *Oprd1*^+^ *gad2*^-^/ *Oprd1*^+^, 15.37% ± 1.57%). (C) Typical images of *gad2*, and *oprm1* expression in the ZIm. (D) Quantitative analyses for the percentage of *Oprm1* or *gad2* expressed neurons in ZIm. (*left*: *gad2*^+^ *Oprm1*^+^ / *gad2*^+^, 25.49% ± 2.79%; *gad2*^+^ *Oprm1*^-^/ *gad2*^+^, 74.51% ± 2.79%; *right*: *Oprm1*^+^ *gad2*^+^ / *Oprm1*^+^, 52.46% ± 3.27%; *Oprm1*^+^ *gad2*^-^/ *Oprm1*^+^, 47.54% ± 3.27%). (E) Typical images of *gad2*, and *oprd1* expression in the ZIm. (F) Quantitative analyses for the percentage of *Oprk1* or *gad2* expressed neurons in ZIm. (*left: gad2*^+^ *Oprk1*^+^ / *gad2*^+^, 26.24% ± 1.81%; *gad2*^+^ *Oprk1*^-^/ *gad2*^+^, 73.76% ± 1.81%; *right*: *Oprk1*^+^ *gad2*^+^ / *Oprk1*^+^, 59.28% ± 2.60%; *Oprk1*^+^ *gad2*^-^/ *Oprk1*^+^, 40.72% ± 2.60%). Scale bars, 20 μm.

**
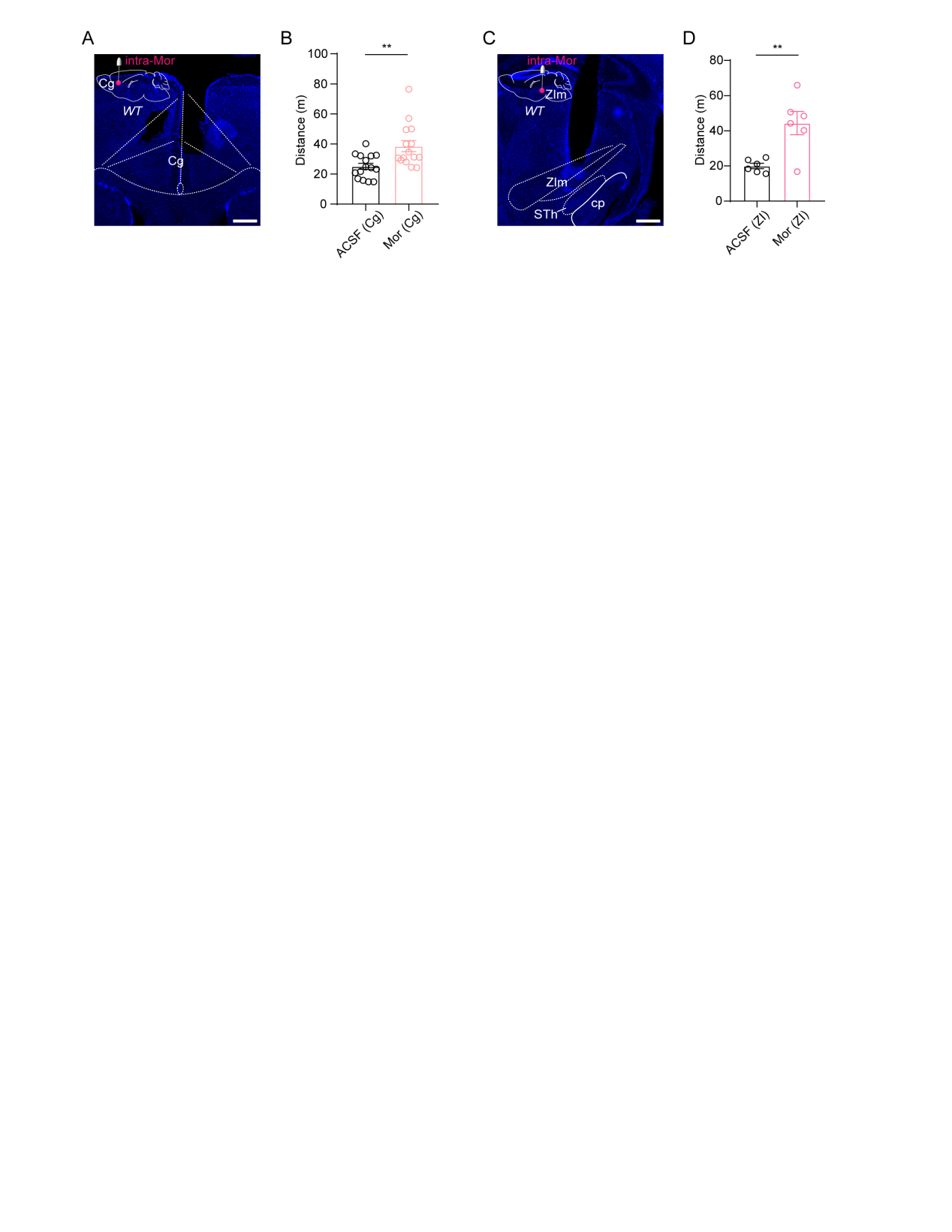
**

**Figure S13. Microinjection of morphine into Cg and ZIm can elicit hyperlocomotion.** (A, C) Schematic of the paradigm of drug administration and representative images of cannula implantation. Scale bar, 200 µm. (B) Statistics of locomotor distance (t_(28)_ = 3.181, ** P = 0.0036). n (mice) = 15 (ACSF-Cg), 15 (morphine-Cg). (D) Statistics of locomotor distance (t_(10)_ = 3.602, ** P = 0.0048). n (mice) = 6 (ACSF- ZIm), 6 (morphine-ZIm). For all figures: two-sided unpaired t-test. ** P < 0.01. Error bars represent s.e.m.


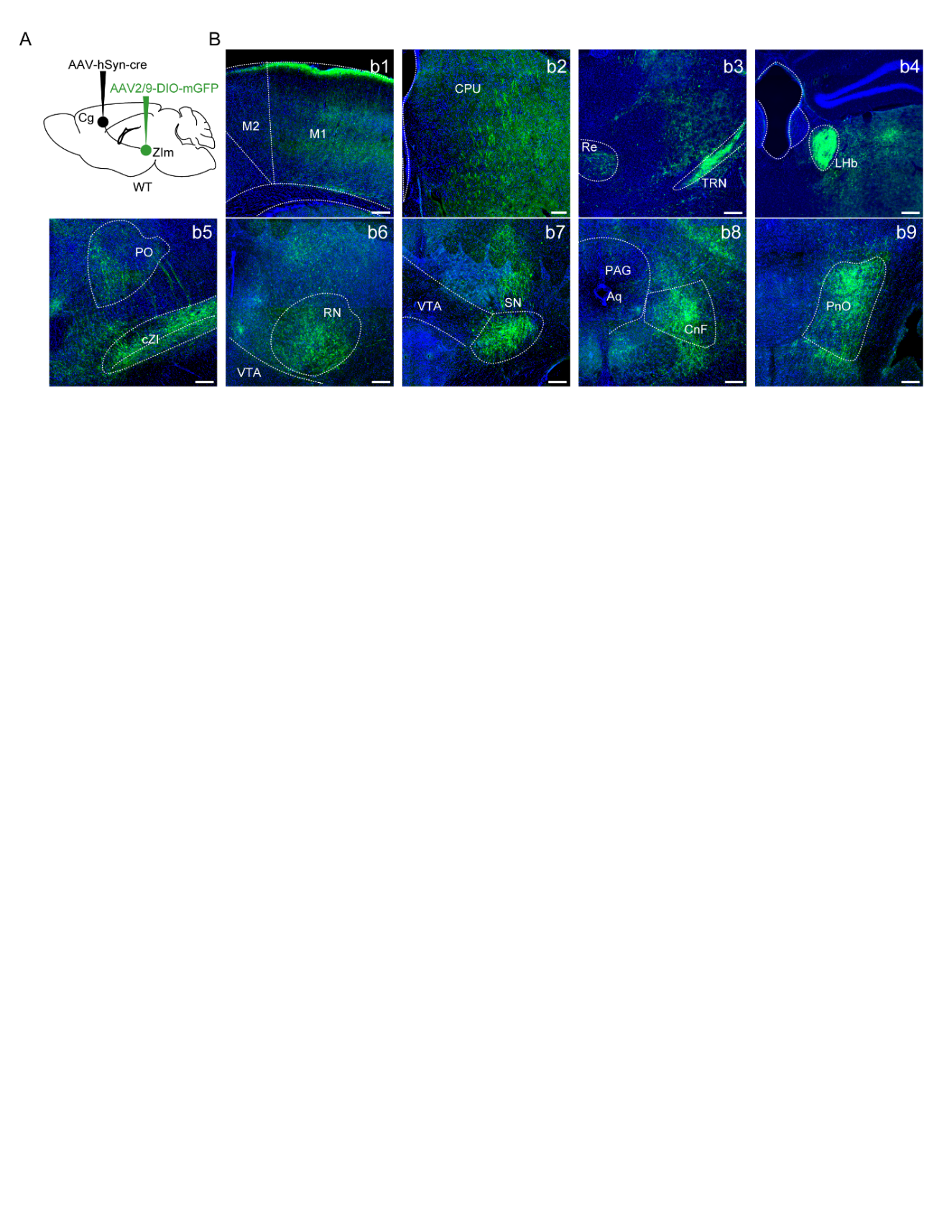


**Figure S14. ZIm^Cg^ project to locomotor-related regions.** (A) Schematic diagram showing injection of AAV-hSyn-Cre and AAV-DIO-mGFP into the Cg and ZIm of WT mice. (B) AAV-DIO-mGFP was injected into the ZIm of WT mice, resulting in labeling of axons in the primary motor cortex (M1), corpus striatum (CPU), thalamic reticular nucleus (TRN), lateral habenula (LHb), posterior nucleus of thalamus (PO), red nucleus (RN), substantia nigra (SN), cuneiform nucleus (CnF), pontine reticular nucleus, oral part (PnO). Scale bar: 100 μm.

Table S1. Extended statistical information for Figures 1-7, and Figures S1-S14.

| Supplementary Table S1 | | | | | | | | | | |
| --- | --- | --- | --- | --- | --- | --- | --- | --- | --- | --- |
| Extended statistical information for Figures 1-7, and Figures S1-S14. | | | | | | | | | | |
| Figure | Experiment | Sample size | | | | | Analysis | P value | t/F/r  value | |
| Fig.2 | | | | | | | | | | |
| Fig.2B | c-Fos+ cells | Saline (3) | | | Morphine (3) | | two-sided unpaired t-test | 0.0141 | t = 4.166, df=4 | |
| Fig.2D | c-Fos+ cells | Saline (3) | | | Morphine (3) | | two-sided unpaired t-test | 0.0387 | t = 3.033,, df=4 | |
| Fig.2G | AUC | Saline (8) | | | Morphine (8) | | two-sided paired t-test | 0.01 | t=3.353, df=8 | |
| Fig.2H | Distance | Saline (6) | | | Morphine (6) | | two-sided paired t-test | 0.0317 | t= 2.386, df=14 | |
| Fig.2M | AUC | Saline (8) | | | Morphine (8) | | two-sided paired t-test | 0.0027 | t=4.534, df=7 | |
| Fig.2N | Distance | Saline (6) | | | Morphine (6) | | two-sided paired t-test | 0.0008 | t=4.247, df=14 | |
| Fig.2I | Correlation of △F/F and Max speed(%) | Saline | | | | | Pearson correlation coefficient | 0.0532 | r=0.8731 | |
| Fig.2J |  | Morphine | | | | |  | 0.0327 | r=0.9086 | |
| Fig.2O |  | Saline | | | | |  | 0.3992 | r=0.4926 | |
| Fig.2P |  | Morphine | | | | |  | 0.0041 | r=0.9215 | |
| Fig.3 | | | | | | | | | | |
| Fig.3C | ACSF vs. CNO vs. Wash | Interaction | | | | | two-sided one-way ANOVA | < 0.0001 | F(1.130, 11.30) = 41.70 | |
|  |  | ACSF (11 cells from 3mice) | CNO (11 cells from 3mice) | | | Wash (11 cells from 3mice) |  | ACSF vs. CNO < 0.0001; | CNO vs. Wash = 0.0066 | |
| Fig.3E | Distance | Interaction | | | | | two-sided one-way ANOVA | < 0.0001 | F(3, 21) = 18.11 | |
|  |  | mCherry+Sal (6) | | | hM4Di+sal (7) | |  | 0.6620 | |  |
|  |  | mCherry+Mor (6) | | | hM4Di+Mor (6) | |  | < 0.0001 | |  |
| Fig.4 | | | | | | | | | | |
| Fig.4B | Body weight score | mCherry+Mor (13) | | | hM4Di+Mor (13) | | two-sided unpaired t-test | 0.343 | t=0.9673, df=24 | |
| Fig.4C | Total distance traveled | mCherry+Mor (13) | | | hM4Di+Mor (13) | | two-sided two-way ANOVA with Sidak test | < 0.0001 | F(5, 120) = 6.365 | |
| Fig.4D | CPP scores | mCherry+Mor (13) | | | hM4Di+Mor (13) | | two-sided unpaired t-test | 0.2248 | t=1.246, df=24 | |
| Fig.4E | Rearing number | mCherry+Mor (6) | | | hM4Di+Mor (6) | |  | 0.1813 | t=1.437, df=10 | |
| Fig.4F | Jump number | mCherry+Mor (6) | | | hM4Di+Mor (6) | |  | 0.9562 | t=0.05637, df=10 | |
| Fig.4G | Hot plate latency | mCherry+Mor (7) | | | hM4Di+Mor (5) | | two-sided two-way ANOVA with Sidak test | 0.5996 | F(2, 20) = 0.5248 | |
| Fig.5 | | | | | | | | | | |
| Fig.5E | Velocity | Control (8) | | | ChR2 (5) | | two-sided two-way ANOVA with Sidak test | < 0.0001 | F(35, 385) = 4.834 | |
| Fig.5F | Distance | Control (8) | | | ChR2 (5) | |  | < 0.0001 | F(2, 22) = 26.96 | |
| Fig.5J | Distance | Control (5) | | | ChR2 (5) | |  | 0.0144 | F(2, 16) = 5.596 | |
| Fig.5N | Distance | Control (6) | | | ChR2 (8) | |  | 0.0390 | F(2, 24) = 3.725 | |
| Fig.5R | Distance | Control (6) | | | ChR2 (6) | |  | 0.2573 | F(2, 20) = 1.454 | |
| Fig.6 | | | | | | | | | | |
| Fig.6F | Distance | Interaction | | | | | two-sided one-way ANOVA with Sidak test | 0.001 | F(3, 30) = 7.016 | |
|  |  | ACSF (5) | | | ICI-174864 (6) | |  | 0.9889 |  | |
|  |  | ACSF + Mor (11) | | | ICI-174864 + Mor (12) | |  | 0.0019 |  | |
| Fig.7 | | | | | | | | | | |
| Fig.7B | expression of *Oprd1* | Scramble (150 cells from 3 mice) | | | DOR-shRNA (328 cells from 5 mice) | | two-sided unpaired t-test | < 0.0001 | t=12.77, df=6 | |
| Fig. 7D | expression of OPRD1 | Scramble (3) | | | DOR-shRNA (3) | |  | 0.0022 | t =7.020, df=4 | |
| Fig.7G | Distance | Interaction | | | | | two-sided one-way ANOVA with Holm-Sidak test | 0.0001 | F(5, 32) = 7.138 | |
|  |  | Scramble+Sal (6) | | | DOR-shRNA+Sal (6) | |  | 0.5073 |  | |
|  |  | Scramble+Mor (6) | | | DOR-shRNA+Mor (6) | |  | 0.0339 |  | |
|  |  | Scramble+Mor+ICI174864 (7) | | | DOR-shRNA+Mor+ICI174864 (7) | |  | 0.8093 |  | |
| Fig. S3 | | | | | | | | | | |
| Fig. S3B | Cg: c-Fos cells | Saline (3) | | Morphine (3) | | | two-sided unpaired t test with Holm-Sidak multiple comparisons | 0.039 |  | |
|  | NAc: c-Fos cells |  |  |  |  |  |  | 0.017 |  |  |
|  | PVT: c-Fos cells |  |  |  |  |  |  | < 0.001 |  |  |
|  | LH: c-Fos cells |  |  |  |  |  |  | 0.001 |  |  |
|  | BLA: c-Fos cells |  |  |  |  |  |  | < 0.001 |  |  |
|  | CeA: c-Fos cells |  |  |  |  |  |  | < 0.001 |  |  |
|  | ZIm: c-Fos cells |  |  |  |  |  |  | 0.014 |  |  |
|  | VTA: c-Fos cells |  |  |  |  |  |  | < 0.001 |  |  |
|  | PAG: c-Fos cells |  |  |  |  |  |  | 0.016 |  |  |
|  | DRN: c-Fos cells |  |  |  |  |  |  | 0.019 |  |  |
| Fig. S4 | | | | | | | | | | |
| Fig.S4B | Distance | Interaction | | | | | two-sided one-way ANOVA with Sidak test | < 0.0001 | F(7, 29) = 24.17 | |
|  |  | mCherry+5mg/kg-Mor (5) | | | hM4Di+5mg/kg-Mor (5) | |  | 0.4376 |  | |
|  |  | mCherry+10mg/kg-Mor (4) | | | hM4Di+10mg/kg-Mor (5) | |  | < 0.0001 |  | |
|  |  | mCherry+15mg/kg-Mor (4) | | | hM4Di+15mg/kg-Mor (6) | |  | < 0.0001 |  | |
| Fig.S4D | Distance | mCherry (5) | | hM4Di (8) | | | two-sided unpaired t-test | 0.3339 | t=1.011, df=11 | |
| Fig.S4E | Open arm time | mCherry (5) | | hM4Di (8) | | |  | 0.6113 | t=0.523, df=11 | |
| Fig.S4F | Close arm distance | mCherry (5) | | hM4Di (8) | | |  | 0.786 | t=0.2783, df=11 | |
| Fig.S4G | Line crossing | mCherry (5) | | hM4Di (8) | | |  | 0.4638 | t=0.759, df=11 | |
| Fig.S4I | CPP scores | mCherry + Mor (7) | | hM4Di + Mor (8) | | |  | 0.1783 | t=1.423, df=13 | |
| Fig.S4K | Hot plate latency | mCherry (8) | | hM4Di (7) | | | two-sided two-way ANOVA with Sidak test | 0.6624 | F(1, 4) = 0.2215 | |
| Fig. S5 | | | | | | | | | | |
| Fig. S5B | Distance | Interaction | | | | | two-sided one-way ANOVA with Sidak test | < 0.0001 | F(3, 21) = 30.38 | |
|  |  | mCherry + Sal (6) | | hM4Di + Sal (7) | | |  | 0.9655 |  | |
|  |  | mCherry + Mor (6) | | hM4Di + Mor (6) | | |  | 0.0014 |  | |
| Fig. S6 | | | | | | | | | | |
| Fig.S6B | Distance | Control (3) | | ChR2 (8) | | | two-sided two-way ANOVA with Sidak test | 0.3962 | F(2, 20) = 0.9700 | |
| Fig. S6D | Distance | Interaction | | | | | two-sided one-way ANOVA with Sidak test | < 0.0001 | F(3, 15) = 34.88 | |
|  |  | mCherry + Sal (5) | | | hM4Di + Sal (5) | |  | 0.1040 |  | |
|  |  | mCherry + Mor (4) | | | hM4Di + Mor (5) | |  | 0.0008 |  | |
| Fig. S6E | Distance | Interaction | | | | | two-sided one-way ANOVA with Sidak test | < 0.0001 | F(3, 19) = 17.30 | |
|  |  | mCherry + Sal (6) | | | hM4Di + Sal (5) | |  | > 0.9999 |  | |
|  |  | mCherry + Mor (6) | | | hM4Di + Mor (6) | |  | 0.0166 |  | |
| Fig. S7 | | | | | | | | | | |
| Fig. S7D | Distance | Interaction | | | | | two-sided one-way ANOVA with Sidak test |  | F(3, 12) = 9.769 | |
|  |  | mCherry + Sal (4) | | | hM4Di + Sal (4) | |  | 0.9623 |  | |
|  |  | mCherry + Mor (4) | | | hM4Di + Mor (4) | |  | 0.8876 |  | |
| Fig. S8 | | | | | | | | | | |
| Fig.S8A | Distance | mCherry + Mor (13) | | hM4Di + Mor (13) | | | two-sided two-way ANOVA with Sidak test | < 0.0001 | F(5, 120) = 10.93 | |
| Fig. S9 | | | | | | | | | | |
| Fig.S9C | Distance | Control (8) | | ChR2 (8) | | | two-sided two-way ANOVA with Sidak test | 0.0109 | F(2, 28) = 5.331 | |
| Fig.S9D | Distance | Control (8) | | ChR2 (8) | | |  | 0.0399 | F(2, 28) = 3.621 | |
| Fig.S9E | Distance | Control (4) | | ChR2 (7) | | |  | < 0.0001 | F(2, 18) = 17.36 | |
| Fig.S9F | Distance | Control (4) | | ChR2 (7) | | |  | 0.008 | F(2, 17) = 6.509 | |
| Fig. S10 | | | | | | | | | | |
| Fig.S10B | RTPP | Control (4) | | ChR2 (6) | | | two-sided unpaired t-test | 0.2343 | t=1.286, df=8 | |
| Fig.S10E | CPP | Control (6) | | ChR2 (6) | | | two-sided unpaired t-test | 0.6628 | t=0.4493, df=10 | |
| Fig.S10F | Distance | Control (8) | | ChR2 (5) | | | two-sided two-way ANOVA with Sidak test | < 0.0001 | F(2, 22) = 20.26 | |
| Fig.S10G | Close arm distance | Control (8) | | ChR2 (5) | | |  | < 0.0001 | F(2, 22) = 21.81 | |
| Fig.S10H | Open arm time | Control (8) | | ChR2 (5) | | |  | 0.0212 | F(2, 22) = 4.618 | |
| Fig.S10I | MBT | Control (8) | | ChR2 (8) | | | two-sided unpaired t-test | 0.2823 | t=1.118, df=14 | |
| Fig.S10J | Digging behavior | Control (6) | | ChR2 (6) | | |  | 0.0735 | t=1.999, df=10 | |
| Fig.S10L | Time of fall off | Control (7) | | ChR2 (13) | | | two-sided two-way ANOVA with Sidak test | 0.4122 | F(1, 18)= 0.7048 | |
| Fig.S10M | Terminal speed | Control (7) | | ChR2 (13) | | |  | 0.3825 | F(1, 18)= 0.8014 | |
| Fig. S11 | | | | | | | | | | |
| Fig.S11G | Distance | Interaction | | | | | two-sided one-way ANOVA with Sidak test | < 0.0001 | F(11, 540) = 4.882 | |
|  |  | ACSF + 5mg/kg-Mor (4) | | | β-FNA + 5mg/kg-Mor (5) | |  | 0.0305 |  | |
|  |  | ACSF + 15mg/kg-Mor (4) | | | ICI-174864 + 15mg/kg-Mor (6) | |  | 0.0280 |  | |
| Fig. S13 | | | | | | | | | | |
| Fig.S13A | Distance | ACSF (15) | | | Mor (15) | | two-sided unpaired t-test | 0.0036 | t=3.181 df=28 | |
| Fig.S13B | Distance | ACSF (6) | | | Mor (6) | |  | 0.0048 | t=3.602, df=10 | |
